# Ligand Field‐Induced Dual Active Sites Enhance Redox Potential of Nickel Hexacyanoferrate for Ammonium Ion Storage

**DOI:** 10.1002/adma.202419446

**Published:** 2025-05-20

**Authors:** Mengmeng Zhou, Tong Wu, Mengde Kang, Tengfei Cheng, Hui Li, Liqing He, Cheng Lian, Tianyi Ma, Qin Zhao

**Affiliations:** ^1^ Key Laboratory for Green Synthesis and Preparative Chemistry of Advanced Materials of Liaoning Province Institute of Clean Energy Chemistry College of Chemistry Liaoning University Shenyang 110036 China; ^2^ Inner Mongolia Engineering Research Centre of Lithium‐Sulfur Battery Energy Storage College of Chemistry and Materials Science Inner Mongolia Minzu University Tongliao 028000 China; ^3^ School of Chemistry and Molecular Engineering East China University of Science and Technology Shanghai 200237 China; ^4^ Hefei General Machinery Research Institute Co., Ltd Hefei 230031 China; ^5^ Centre for Atomaterials and Nanomanufacturing (CAN) School of Science RMIT University Melbourne Victoria 3000 Australia

**Keywords:** aqueous ammonium ion batteries, dual active sites, high redox potential, prussian blue analogues

## Abstract

Enhancing the redox potential of cathode materials is vital for increasing the energy density of aqueous ammonium ion batteries (AIBs). Prussian blue analogues (PBAs), with their inherently high redox potentials and open frameworks, are promising candidates. However, further boosting their redox potential and understanding their NH_4_^+^ ion storage mechanisms remain critical challenges. In this work, a novel ligand field‐induced dual active sites mechanism is introduced by incorporating Ni into the PBA framework, activating Ni as an additional redox center for NH_4_^+^ ion storage. The electron transfer from Ni to Fe within the Ni─N≡C─Fe chain enhances the redox potential and electrochemical performance of nickel hexacyanoferrate (NiHCF). For the first time, the electrochemical activity of Ni is demonstrated in NiHCF during NH_4_^+^ ions intercalation and de‐intercalation. NiHCF exhibits elevated redox potentials, superior rate performance, and robust cycling stability compared to iron hexacyanoferrate (FeHCF). Advanced characterization techniques and density functional theory calculations confirm the activation of Ni and the enhanced interaction between NH_4_^+^ ions and the framework. These findings provide new insights into the NH_4_^+^ ion storage mechanism of PBAs and offer a promising strategy for designing high‐energy‐density cathode materials for AIBs.

## Graphical abstract

The work aims to explore the electrochemical performance and energy storage mechanism of nickel hexacyanoferrate (NiHCF) in ammonium ion batteries (AIBs). The effect of ligand field‐induced dual active sites has been revealed for the first time. Ni atoms of NiHCF display electrochemical activity in AIBs. Additionally, Ni can balance the charge distribution and further enhance the redox potential.

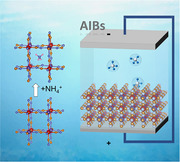

## 1 Introduction

As industrial advancements continue to escalate, the human demand for rechargeable batteries is ever‐increasing. Currently, due to high energy density and power density, lithium‐ion batteries are the predominant choice for large‐scale electrical applications.^[^ ^1^ ^]^ However, their widespread adoption is hindered by high costs and significant safety concerns.^[^ ^2^, ^3^ ^]^ To satisfy the increasing demand of human society, there has been a significant shift toward the development of green, sustainable, and eco‐friendly aqueous rechargeable batteries.^[^ ^4^, ^5^ ^]^ Among these, aqueous ammonium ion batteries (AIBs) have emerged as a superior option due to the superiority of their safety, efficiency, and cost‐effectiveness.^[^ ^6^, ^7^, ^8^ ^]^ AIBs leverage the advantageous properties of NH_4_^+^, including the small hydrated ionic radii (3.31 Å), low molar mass (18 g mol^−1^), and flexible tetrahedron structure. These features enable AIBs to exhibit high‐rate performance and substantial capacity, making them a compelling alternative for energy storage applications.^[^ ^6^, ^9^, ^10^, ^11^, ^12^, ^13^ ^]^

Current research on electrode materials for AIBs encompasses a variety of substances, including metal oxides, metal sulfides, organic materials, and notably, Prussian blue and its analogues (PBAs).^[^ ^10^, ^14^, ^15^, ^16^, ^17^ ^]^ PBAs, with the general formula KM[Fe(CN)_6_] (where M denotes a transition metal such as Fe, Ni, Cu, Co, etc.) are recognized as promising cathode materials due to their open 3D frameworks facilitating ion diffusion, and their robust structural stability.^[^ ^18^, ^19^, ^20^, ^21^, ^22^ ^]^ However, a critical limitation of PBAs in AIB applications is their relatively low redox potential, which constrains the achievable energy density of the batteries. Therefore, enhancing the redox potential of these compounds is crucial for advancing AIBs technology to meet the growing energy demands.^[^ ^23^, ^24^, ^25^ ^]^ Transition metal substitution within the PBA framework has been explored as a prevalent strategy to modulate their electrochemical properties and enhance performance.^[^ ^26^, ^27^, ^28^ ^]^ For instance, iron hexacyanoferrate achieves a working potential of 0.36/0.34 V (vs Ag/AgCl), whereas nickel hexacyanoferrate exhibits a heightened working potential of 0.58/0.55 V (vs Ag/AgCl).^[^ ^29^, ^30^, ^31^, ^32^ ^]^ Moreover, Ni substitution can modify the electronic structure of PBAs through ligand field effects, influencing the charge distribution and redox activity within the framework.^[^ ^33^ ^]^ In previous reports, substituting Ni could induce strong charge‐spin‐lattice coupling between Ni─N and Fe─C, activate the redox couples, optimize the charge distribution of the framework, and further enhance the cycling performance in metal ion batteries.^[^ ^34^, ^35^ ^]^ Additionally, Ni substitution has been reported to enhance the structural stability of PBAs, further achieving increased cycling performance. Despite these advancements, the underlying mechanisms by which Ni substitution enhances the redox potential and affects the NH_4_^+^ storage behavior in AIBs remain inadequately understood.^[^ ^36^ ^]^ Particularly, there is a lack of comprehensive studies explaining how Ni substitution influences the electronic structure and operating mechanism during cycling. Moreover, previous reports have often considered Ni to be electrochemically inert in AIBs, primarily attributing the redox activity to Fe centers, without providing sufficient experimental evidence to verify the role of Ni.^[^ ^31^, ^32^ ^]^ This gap in understanding hinders the rational design of high‐performance PBA cathodes for AIBs.

In this work, we aim to address these challenges by systematically investigating the implications of Ni substitution in nickel hexacyanoferrate (NiHCF) for NH_4_^+^ ion storage in AIBs. We hypothesize that Ni substitution activates Ni as an additional redox center through ligand field‐induced electron transfer, leading to enhanced redox potential and improved electrochemical performance. For the first time, our study reveals the electrochemical activity of Ni in NiHCF during NH_4_^+^ intercalation and de‐intercalation processes. We introduce the concept of a ligand field‐induced dual active sites mechanism, wherein the ligand field modulation caused by Ni substitution triggers electron transfer from Ni^2^⁺ to Fe^3^⁺ within the Ni─N≡C─Fe chain. This electron redistribution activates Ni as an additional redox center alongside Fe, creating synergistic dual active sites that enhance the redox potential and overall electrochemical performance of NiHCF in AIBs.

Our findings demonstrate that NiHCF achieves higher redox potentials of 0.45/0.51 V and 0.63/0.67 V (vs SCE), compared to the lower redox potential of 0.22/0.25 V (vs SCE) observed in iron hexacyanoferrate (FeHCF). Furthermore, NiHCF obtains a capacity of 61.3 mAh g^−1^ at 50 mA g^−1^, superior rate performance, and robust cycling stability with a capacity retention of 71.1% after 1000 cycles. The enhanced performance is attributed to the synergistic effects of the dual active sites and the optimized charge distribution within the framework, resulting from the ligand field‐induced electron transfer. Ex situ characterizations confirm the reversible structural variations during charging and discharging processes, providing a comprehensive understanding of the mechanisms at play. By linking these findings back to the ligand field‐induced dual active sites mechanism, we offer insights into the structural and electronic factors contributing to the enhanced performance. Our study not only confirms the electrochemical participation of Ni in the NH_4_^+^ ion storage process but also elucidates the principle behind the role of Ni substitution in increasing the redox potential. By revealing the higher adsorption energy between NH_4_^+^ and N atoms in NiHCF, we provide insights into the structural and electronic factors contributing to the enhanced performance. This work offers a pivotal guide for future research on PBAs and opens new avenues for designing high energy density cathode materials for AIBs.

## 2 Results and Discussion

### 2.1 Materials and Characterizations

**Figure 1:**
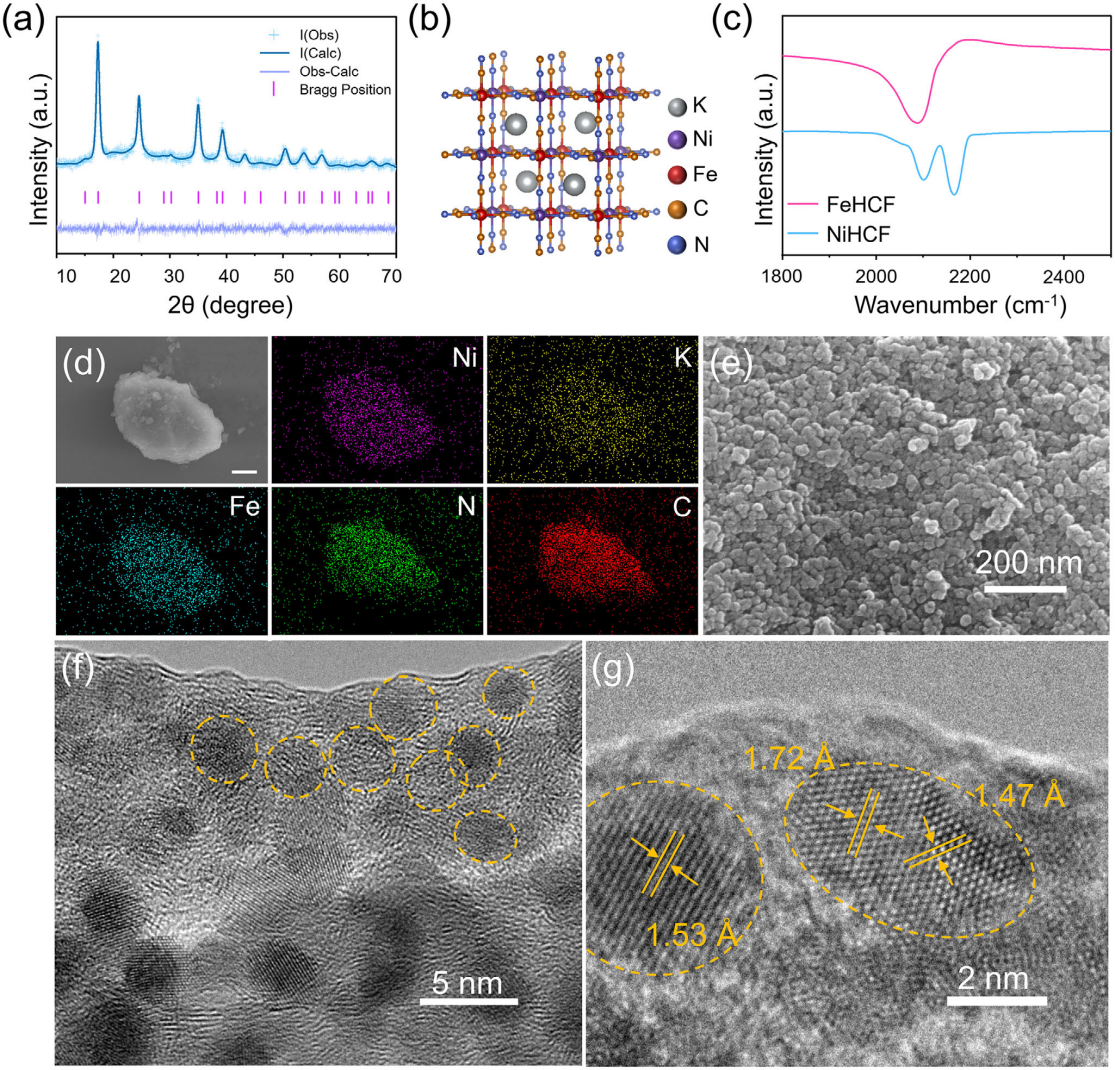
Structure and morphology characterization of NiHCF and FeHCF. a) The XRD pattern and Rietveld refinement of NiHCF (R_wp_ = 3.739%). b) Structural diagram illustrating the atomic arrangement within NiHCF. c) FTIR patterns of NiHCF and FeHCF. d) SEM image and its EDS element mapping images with a scale of 3 µm. e) SEM image of NiHCF. f,g) TEM and HR‐TEM images of NiHCF.

Ni‐substituted NiHCF was prepared via a facile co‐precipitation method at room temperature, using Ni^2+^ and Fe(CN)_6_^3−^ as precursors. For comparison, FeHCF was synthesized similarly to serve as a comparative reference for evaluating the impact of Ni substitution on NH_4_^+^ storage (Figure S1, Supporting Information). X‐ray diffraction (XRD) patterns of NiHCF and FeHCF (**Figure** **1**a; Figure S2, Supporting Information) were analyzed using Rietveld refinement to determine their crystallographic properties, with detailed results provided in Tables S1 and S2 (Supporting Information). The refinement confirms that both NiHCF and FeHCF crystallize in the face‐centered cubic phase within the Fm‐3m space group.^[^ ^37^ ^]^ No diffraction peaks relating to impurities were detected. As shown in Figure 1b, NiHCF features an open 3D framework constructed by FeC_6_ octahedrons and NiN_6_ octahedrons.^[^ ^38^ ^]^ Similarly, the FeHCF framework comprises low‐spin FeC_6_ octahedrons and high‐spin FeN_6_ octahedrons (Figure S3, Supporting Information).^[^ ^18^ ^]^ According to Fourier transform infrared (FTIR) spectra (Figure 1c), the peaks at 2104 and 2164 cm^−1^ are assigned to the stretching vibration of Ni─N≡C─Fe^3+^ and Ni─N≡C─Fe^2+^ for NiHCF, while the peak at 2086 cm^−1^ attributes to the stretching vibration of Fe─C≡N─Fe for FeHCF.^[^ ^39^, ^40^ ^]^ Furthermore, Raman spectrum (Figure S4, Supporting Information) show the characteristic peaks at 2192 and 2132 cm^−1^ for NiHCF, assigned to the C≡N stretching vibrations of Fe─C≡N─Ni, while FeHCF exhibits characteristic peaks at 2154 and 2095 cm^−1^ corresponding to Fe─C≡N─Fe vibrations.^[^ ^41^, ^42^ ^]^ These XRD, FTIR, and Raman results confirm the successful synthesis of NiHCF and FeHCF, with Ni substitution having no detectable impact on the overall structure of the PBA framework.

Thermogravimetric analysis (TGA) provides insight into the crystal water content of NiHCF and FeHCF (Figure S5, Supporting Information), showing weight losses below 200 °C due to absorbed water and between 200–300 °C from crystal water.^[^ ^43^ ^]^ NiHCF and FeHCF have similar crystal water contents of 12.2% and 12.8%, respectively, suggesting comparable crystallinity and implying that the presence of crystal water content is unlikely to significantly influence their electrochemical performance. Inductively coupled plasma‐optical emission spectrometry (ICP‐OES) results (Table S3, Supporting Information) confirm the stoichiometric ratios of Ni and Fe. According to TGA and ICP‐OES analyses, the chemical formulas of NiHCF and FeHCF are determined as K_0.136_Ni[Fe(CN)_6_]_0.65_·1.51H_2_O and K_0.178_Fe[Fe(CN)_6_]_0.49_·1.36H_2_O, respectively. Scanning electron microscope (SEM) images reveal that both NiHCF and FeHCF display massive morphology, while the energy dispersive spectrometer (EDS) elemental mapping confirms the uniform distributions of Ni, K, Fe, N, and C in NiHCF, and C, N, K, and Fe in FeHCF (Figure 1d; Figure S6, Supporting Information). The detailed element compositions for NiHCF and FeHCF are listed in Tables S4 and S5 (Supporting Information). Furthermore, the massive structure consists of uniformly sized nanoparticles (Figure 1e; Figure S7, Supporting Information), with statistical analysis displaying average particle sizes of 21 and 19 nm for NiHCF and FeHCF, respectively (Figure S8a,b, Supporting Information). Nanoparticles of NiHCF are formed by the aggregation of smaller grains, which may mitigate side reactions between the material and the electrolyte (Figure 1f). High‐resolution transmission electron microscopy (HR‐TEM) images further reveal the lattice fringes corresponding to specific crystal planes in both NiHCF and FeHCF. For NiHCF, the lattice spacings of 1.53, 1.72, and 1.47 Å ascribe to the (311), (300), (222) planes, respectively (Figure 1g). In FeHCF, the lattice spacing of 3.60 and 1.53 Å correspond to the (110) and (311) planes, respectively (Figure S9, Supporting Information). No grains similar to those observed in NiHCF were detected in FeHCF, which may be attributed to a slower co‐precipitation reaction between Ni^2+^ and Fe(CN)_6_^3−^. These results indicate that the synthesized NiHCF and FeHCF share similar chemical structure and morphological characteristics, which suggests that structural differences are unlikely to account for the electrochemical performance variations between NiHCF and FeHCF. These morphological and structural similarities suggest that the differences in electrochemical performance between NiHCF and FeHCF are not due to variations in physical structure but are likely attributed to electronic effects arising from Ni substitution.

**Figure 2:**
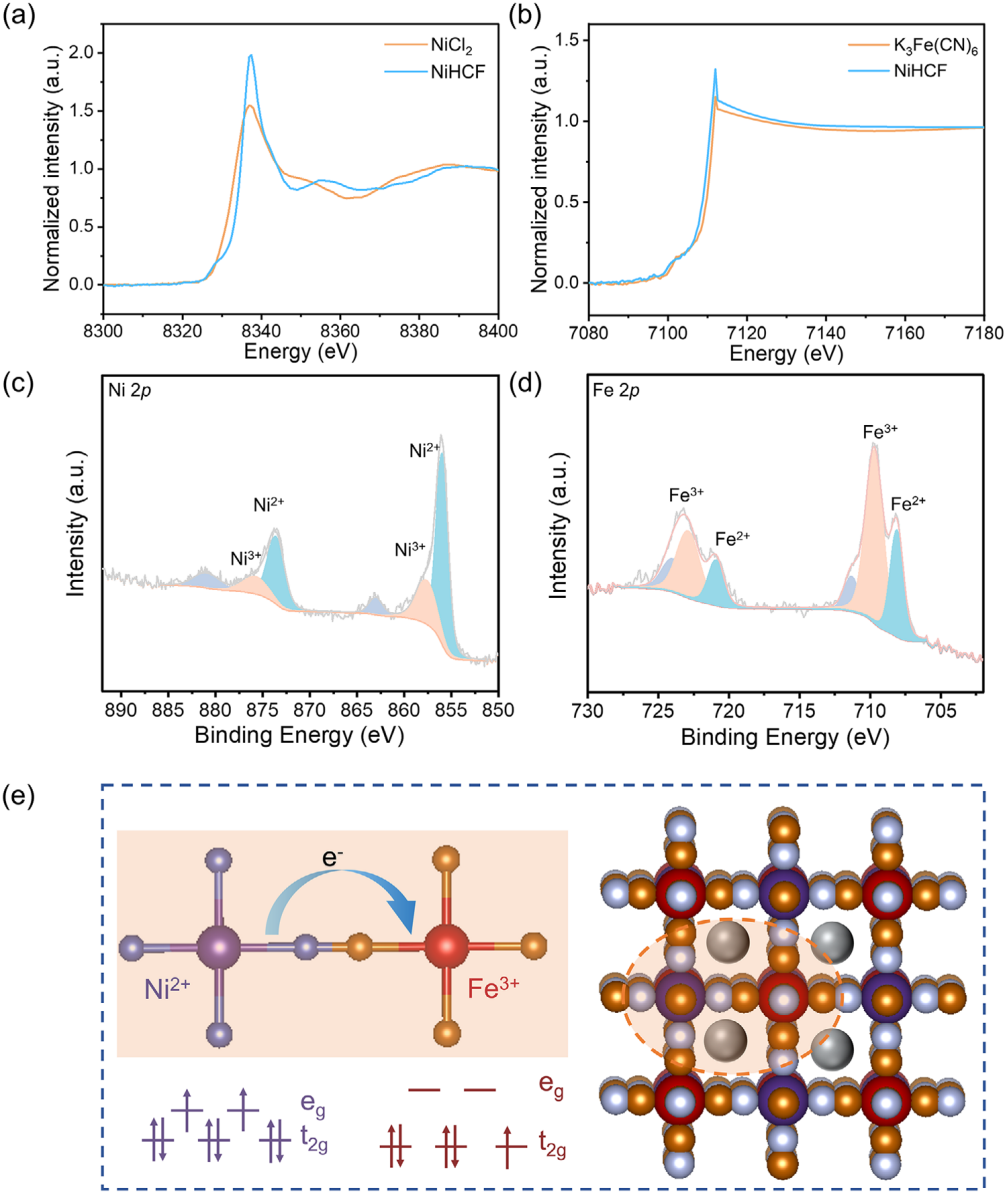
a) Ni K‐edge XANES spectra comparing NiHCF and NiCl_2_, which serves as one of the precursors in the co‐precipitation synthesis. b) Fe K‐edge XANES spectra comparing K_3_Fe(CN)_6_ and NiHCF, highlighting the Fe component from the reaction precursor K_3_Fe(CN)_6_. c,d) The XPS spectra of NiHCF focusing on Ni 2*p* and Fe 2*p* orbits. e) Schematic representation of the 3*d* orbitals for Ni^2+^ and Fe^3+^ under the octahedral crystal field.

To investigate the valence states of Ni and Fe and the influence of Ni substitution on charge distribution within NiHCF, X‐ray absorption near edge structure (XANES) and X‐ray photoelectron spectroscopy (XPS) were employed. The Ni K‐edge XANES spectra reveal a positive shift in the absorption edge of NiHCF toward higher binding energy compared to NiCl_2_ (**Figure** **2**a), while the Fe K‐edge XANES spectra show a shift toward lower binding energy compared to K_3_Fe(CN)_6_ (Figure 2b). As these reference compounds serve as raw materials for synthesizing NiHCF, this observation suggests that the valence state of Ni in NiHCF is greater than +2, while the valence state of Fe is less than +3. These shifts indicate potential electron transfer between Ni and Fe along the C≡N chain during the co‐precipitation process of NiHCF.^[^ ^44^, ^45^, ^46^ ^]^ XPS analysis provides further insights into the electronic structure. The XPS full spectra (Figure S10, Supporting Information) reveal the presence of K, Ni, Fe, C, N, and O elements in NiHCF and K, Fe, C, N, and O elements in FeHCF. In the Ni 2*p* XPS spectrum of NiHCF (Figure 2c), the peaks at binding energies of 856.0 and 873.6 eV are assigned to 2*p*_1/2_ and 2*p*_3/2_ of Ni^2+^, while the peaks at binding energies of 857.7 and 875.3 eV correspond to the 2*p*_1/2_ and 2*p*_3/2_ of Ni^3+^.^[^ ^47^, ^48^ ^]^ Additionally, in the Fe 2*p* XPS spectrum of NiHCF (Figure 2d), the peaks at 708.1 and 721.0 eV are assigned to 2*p*_1/2_ and 2*p*_3/2_ of Fe^2+^, and the peaks at 709.7 and 723.0 eV are attributed to Fe^3+^.^[^ ^39^, ^49^ ^]^ By calculating the integral areas of these characteristic peaks in the Ni 2*p* and Fe 2*p* XPS spectra, the ratios of Ni^2+^ to Ni^3+^ and Fe^3+^ to Fe^2+^ were found to be 2.51 and 2.53, indicating average valence states of Ni (+2.23) and Fe (+2.72) in NiHCF. In contrast, FeHCF exhibits an average Fe valence state of 2.29 (Figure S11, Supporting Information).^[^ ^39^ ^]^ These findings strongly suggest Ni substitution induces electron transfer from Ni^2+^ to Fe^3+^ within the Ni─N≡C─Fe chain, resulting in partial oxidation of Ni and reduction of Fe. This ligand field‐induced electron transfer alters the electronic structure and charge distribution within the framework, activating Ni as an additional redox center. According to crystal field theory, the 3*d* orbits of Fe^3+^ and Ni^2+^ are split into e_g_ and t_2g_ orbitals under the octahedral coordination environment (Figure 2e). Here, the C in C≡N chain donates electrons to Fe^3+^, stabilizing Fe^3+^ in a low‐spin state, while the N in C≡N presents an electron‐withdrawing effect on Ni^2+^, facilitating partial 3*d* electron transfer from Ni to Fe through the C≡N ligand. Notably, Ni^2+^ is not fully oxidized to Ni^3+^, as the complete oxidation would result in an outer electron configuration (4*s*_2_3*d*_7_) prone to Jahn–Teller distortion in NiN_6_ octahedron (Figure S12, Supporting Information).^[^ ^50^ ^]^ This partial electron transfer balances the charge distribution without compromising the structural integrity of NiHCF. Additionally, the enhanced electron‐withdrawing effect of the N atoms connected to Ni upon electron loss strengthens the framework stability. The activation of Ni, in conjunction with Fe, creates synergistic dual active sites that contribute to the increased redox potential and improved electrochemical performance observed in NiHCF.

### 2.2 Electrochemical Characterizations

**Figure 3:**
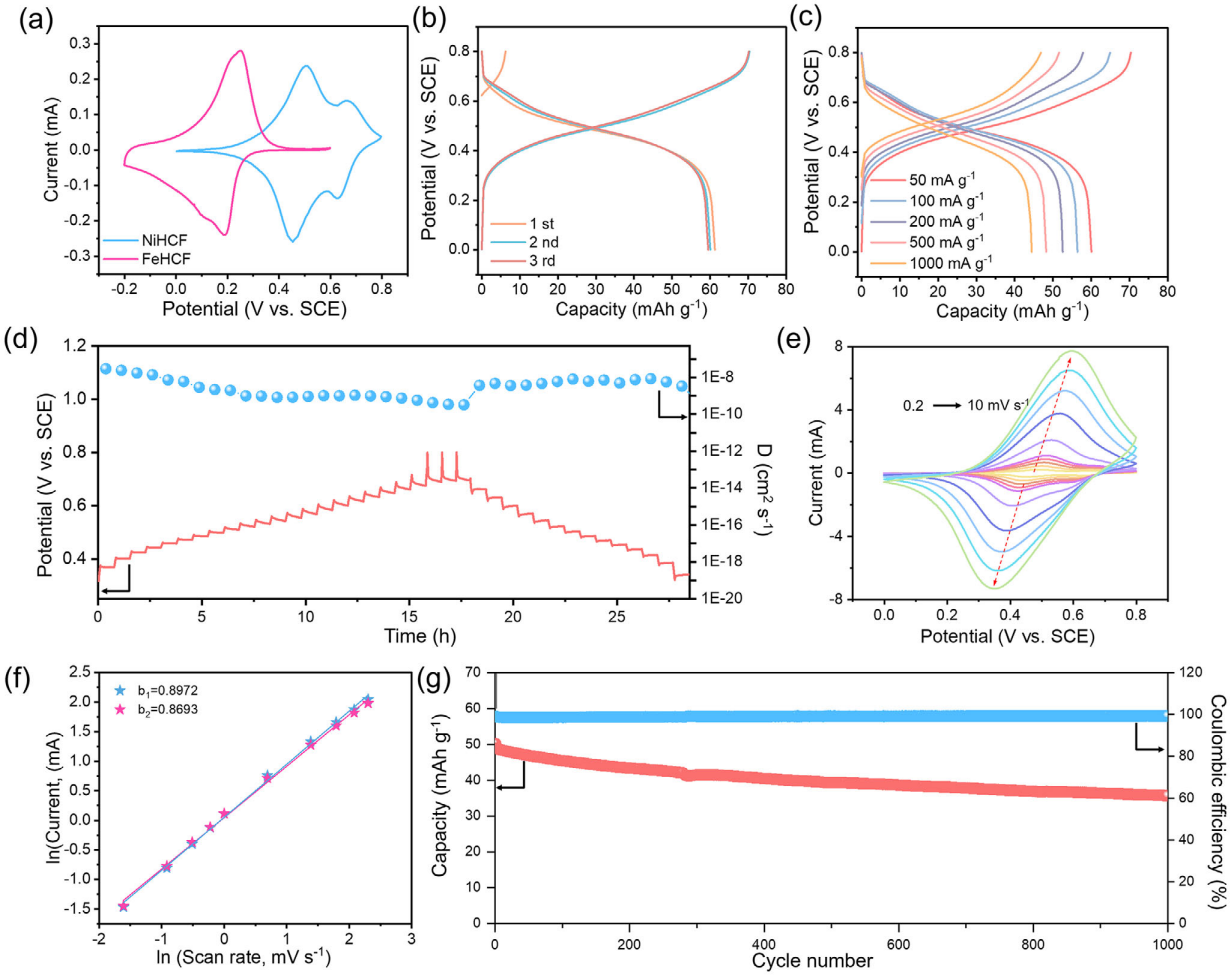
The electrochemical performance of NiHCF in 2 m ammonium acetate. a) CV curves of NiHCF and FeHCF at 0.2 mV s^−1^, with potential window of 0–0.8 V and −0.2–0.6 V (vs SCE), respectively. b) The first three galvanostatic charging and discharging profiles at a current density of 50 mA g^−1^. c) The galvanostatic charging and discharging profiles at different current densities of 50, 100, 200, 500, and 1000 mA g^−1^. d) GITT curves and the relevant NH_4_^+^ ion diffusion coefficients. e) CV profiles at scan rates of 0.2, 0.4, 0.6, 0.8, 1.0, 2.0, 4.0, 8.0, and 10 mV s^−1^. f) The “b” values are derived from the redox peaks, with “b_1_” and “b_2_” representing oxidation peaks and reduction peaks in (e). g) The cycling performance over 1000 cycles at a current density of 1000 mA g^−1^.

To elucidate the NH_4_^+^ storage behavior and validate the ligand field‐induced dual active sites mechanism in NiHCF, we conducted comprehensive electrochemical characterizations and compared the results with FeHCF. As shown in **Figure** **3**a, the cyclic voltammetry (CV) curve of NiHCF in AIBs displays two distinct pairs of symmetric redox peaks at 0.45/0.51 V and 0.63/0.67 V (vs SCE). The former pair is attributed to the redox couple of Fe^2+^/Fe^3+^, while the latter is associated with the redox couple of Ni^2+^/Ni^3+^.^[^ ^51^, ^52^ ^]^ Despite prior studies suggesting the electrochemical inactivity of Ni in NiHCF, our work reveals its participation in redox reactions in AIBs.^[^ ^31^, ^32^, ^51^ ^]^ The participation of Ni in the redox process supports the existence of synergistic dual active sites resulting from ligand field‐induced electron transfer. To further substantiate the activation of Ni, a CV test of NiHCF was carried out in aqueous potassium ion batteries. Interestingly, in this environment, NiHCF exhibited only the Fe^2+^/Fe^3+^ redox peaks at 0.47/0.48 V (vs SCE) without any noticeable Ni^2+^/Ni^3+^ activity (Figure S13, Supporting Information). This suggests that the interaction between NH_4_^+^ ions and the N atoms in NiHCF is crucial for activating the electrochemical behavior of Ni. The hydrogen bonding between NH_4_^+^ and the N atoms may facilitate the ligand field modulation, promoting electron transfer from Ni^2+^ to Fe^3+^ and activating Ni as a redox center. In contrast, the CV curve of FeHCF displays a single pair of redox peaks at 0.19/0.25 V (vs SCE), attributable to the Fe^2+^/Fe^3+^ couple.^[^ ^40^ ^]^ Notably, the Fe^2+^/Fe^3+^ redox potential in NiHCF is higher than that in FeHCF, which indicates that Ni substitution and the resulting ligand field effects influence the redox potential of the Fe centers. The elevated redox potential can be attributed to the altered electronic environment and enhanced interaction between NH₄⁺ ions and the framework, due to the ligand field‐induced electron transfer.

Galvanostatic charging and discharging tests of NiHCF and FeHCF have been performed at a current density of 50 mA g^−1^ to evaluate the practical electrochemical performance (Figure 3b; Figure S14, Supporting Information). NiHCF obtains discharging capacities of 61.3, 60.1, and 59.5 mAh g^−1^ over the initial three cycles, with a stable discharging platform at approximately 0.45 V (vs SCE). In comparison, FeHCF delivers higher discharging capacities of 89.6, 86.9, 85.7 mAh g^−1^ with a lower discharge platform of 0.18 V (vs SCE). The reduced capacity of NiHCF can be attributed to the fewer electrons contributed by Ni for NH_4_^+^ ion storage compared to Fe in FeHCF. Despite its constrained theoretical capacity, NiHCF delivers a higher practical capacity than other reported PBAs (Table S6, Supporting Information).^[^ ^53^ ^]^ The rate performance of NiHCF and FeHCF is shown in Figure S15 (Supporting Information). With the current density increase from 50 to 1000 mA g^−1^, both NiHCF and FeHCF display decreasing capacities but fully recover their original capacities when the current density returns to 50 mA g^−1^. NiHCF achieves discharging capacities of 60.1, 56.4, 52.6, 48.3, 44.5 mAh g^−1^ at the current densities of 50, 100, 200, 500, 1000 mA g^−1^ (Figure 3c). In comparison, FeHCF achieves discharging capacities of 86.9, 78.8, 72.9, 63.8, 55.1 mAh g^−1^ at the current densities of 50, 100, 200, 500, 1000 mA g^−1^ (Figure S16, Supporting Information). At 1000 mA g^−1^, NiHCF maintained 74.0% of its initial capacity at 50 mA g^−1^, compared to 63.4% for FeHCF, indicating that NiHCF presented superior rate performance. The superior rate performance of NiHCF can be attributed to the enhanced electron transfer kinetics and ion diffusion facilitated by the dual active sites and optimized charge distribution within the framework.

To further investigate the kinetics of NH_4_^+^ ion storage, we employed the galvanostatic intermittent titration technique (GITT) to evaluate the NH_4_^+^ ions diffusion coefficient (D) in NiHCF and FeHCF (Figure 3d; Figure S17, Supporting Information). The calculated D values for both NiHCF and FeHCF range from 10^−10^ to 10^−8^ cm^2^ s^−1^. Notably, within the charging and discharging plateaus, NiHCF exhibits slightly higher D values than FeHCF, indicating a more rapid NH_4_^+^ diffusion rate in NiHCF. This enhancement is consistent with the proposed mechanism, where the ligand field‐induced activation of Ni facilitates ion diffusion through the framework. Electrochemical impedance spectroscopy measurements provided additional insights into the charge transfer processes (Figure S18, Supporting Information). NiHCF displays a lower charge transfer resistance (R_ct_) compared to FeHCF, suggesting more efficient electron transfer and faster electrochemical reactions. The decreased R_ct_ in NiHCF can be attributed to the synergistic effect of the dual active sites, which enhances conductivity and facilitates charge transfer during NH₄⁺ intercalation and de‐intercalation.^[^ ^54^ ^]^ The CV curves of NiHCF and FeHCF, illustrated in Figure 3e and Figure S19a (Supporting Information), are recorded at scan rates from 0.2 to 10 mV s^−1^. With the increasing scan rates, both the peak current and voltage polarization increased, while the overall shape of CV curves remained consistent. The relationship between scan rate (*v*) and peak currents (*i*) is described by Equation S2 (Supporting Information). For NiHCF, the derived *b* values for peak 1 and peak 2 are 0.897 and 0.869 (Figure 3f), whereas for FeHCF, these values are 0.778 and 0.881 (Figure S19b, Supporting Information), implying that NH_4_^+^ storage in both materials is governed by a mix of diffusive and capacitive processes. Specific capacitive behavior contributions of NiHCF and FeHCF were calculated using Equation S3 (Supporting Information). With scan rates increasing from 0.2 to 10 mV s^−1^, the capacitive‐controlled NH_4_^+^ storage proportion in NiHCF increased from 80.5% to 99.7% (Figure S20a, Supporting Information), while in FeHCF, it increased from 60.5% to 89.4% (Figure S20b, Supporting Information). This higher capacitive contribution in NiHCF aligns with superior rate performance. Long‐term cycling tests at a high current density of 1000 mA g^−1^ demonstrate the excellent cycling stability of NiHCF (Figure 3g; Figure S21, Supporting Information). After 600 cycles, NiHCF retains 76.9% of its initial capacity, while FeHCF retains 72.2%, with both materials exhibiting ≈100% coulombic efficiency. NiHCF also achieves 71.1% capacity retention after 1000 cycles, revealing the longer cycling lifespan (Figure 3g). Additionally, Figure S22 (Supporting Information) reveals that the capacity retention of NiHCF is higher than that of FeHCF for 5, 10, 50, 100, and 600 cycles. The improved cycling performance of NiHCF can be attributed to the structural stability provided by the ligand field‐induced electron redistribution and the synergistic effect of the dual active sites. The activation of Ni not only enhances the redox potential but also contributes to maintaining the integrity of the framework during repeated cycling. Compared with other materials, NiHCF in this work exhibits a higher redox potential and is expected to obtain higher energy density and power density in AIBs (Figure S23, Supporting Information). Moreover, NiHCF demonstrates a notably higher practical‐to‐theoretical capacity ratio (85.6%), higher working potential (0.45 V vs SCE), and longer cycling performance (71.1% after 1000 cycles) relative to other reported cathode materials (Figure S24 and Table S7, Supporting Information). In summary, the electrochemical characterization results confirm that NiHCF demonstrates elevated redox potential, robust rate capability, and stable cycling performance compared to FeHCF, positioning it as a promising cathode material for AIBs.

### 2.3 Ammonium Ion Storage Mechanism

**Figure 4:**
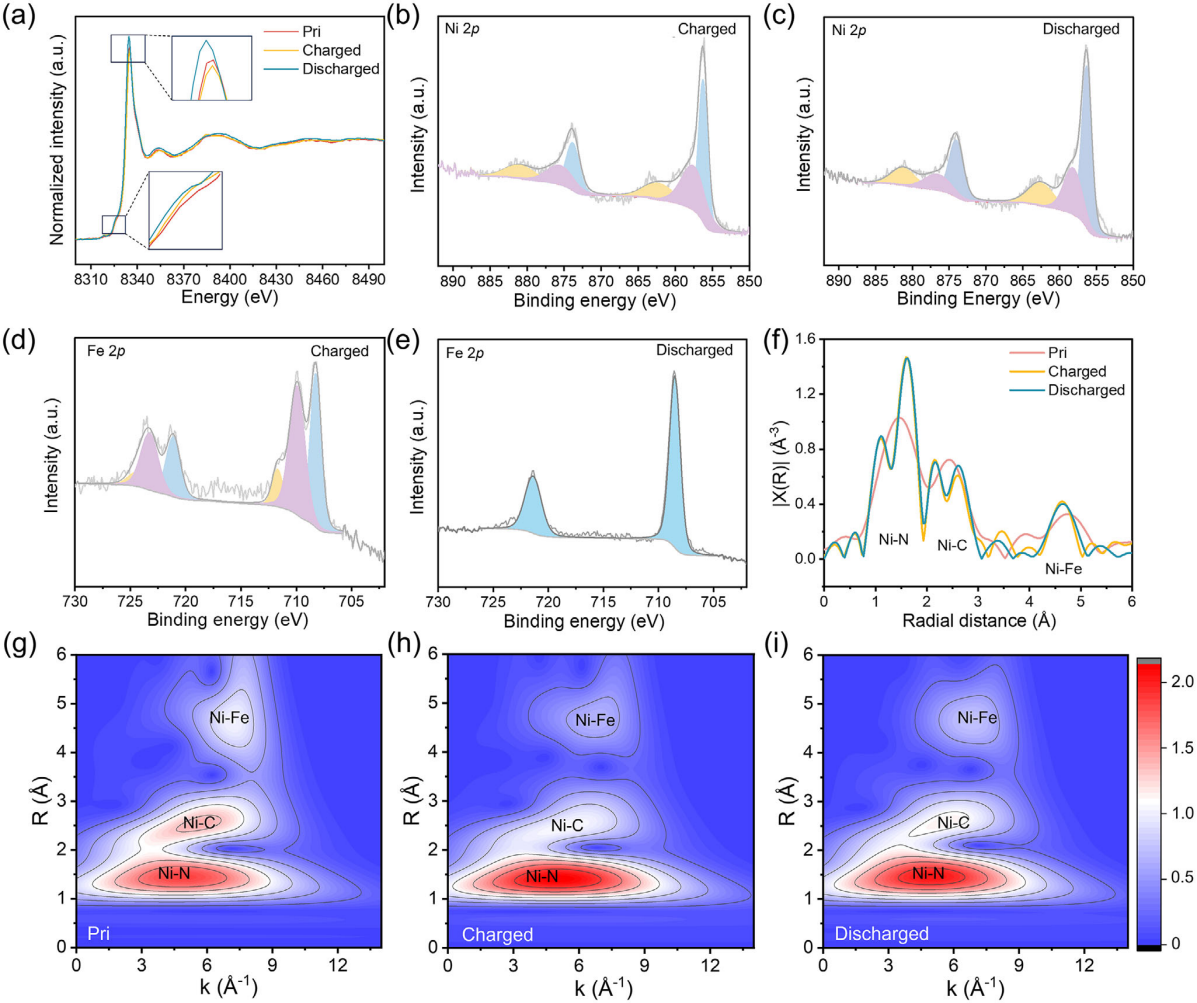
Mechanism discussion of electron transfer and structural variation in NiHCF. a) Ni K‐edge XANES spectra at pristine, charged, and discharged states. b) Ni 2*p* XPS spectrum at the charged state. c) Ni 2p XPS spectrum at the discharged state. d) Fe 2*p* XPS spectrum at the charged state. e) Fe 2*p* XPS spectrum at the discharged state. f) Ni K‐edge EXAFS spectra R‐space curves at pristine, charged, and discharged states, showing atomic distance variations. g–i) Ni K‐edge wavelet transform EXAFS maps at pristine, charged, and discharged states, respectively, highlighting the structural evolution of Ni─N, Ni─C, and Ni─Fe bonds during the cycling process.

To elucidate the electron transfer mechanism and structural variations occurring in NiHCF during the charging and discharging process, X‐ray absorption spectroscopy (XAS) and XPS analyses were conducted. **Figure** **4**a illustrates the Ni K edge XANES spectra of NiHCF in its pristine state (Pri), charged to 0.8 V (vs SCE) state (Charged), and discharged to 0 V (vs SCE) state (Discharged). The noticeable shift of the Ni K‐edge to lower energy in the charged state compared to the discharged state indicates that the valence state of Ni is higher in a charged state.^[^ ^44^ ^]^ This observation confirms that electrochemical activity involving Ni atoms occurs during electrochemical cycling, highlighting the active role of Ni in NH_4_^+^ ion storage. Notably, the K‐edge binding energy of Ni in its pristine state is higher than that in the discharged state, indicating a higher oxidation state of Ni in the pristine state compared to that in a discharged state. This finding suggests that Ni participates in NH_4_^+^ storage during the discharging process, and the discharging process attenuates the electron transfer interactions between Ni and Fe. XPS spectra further explored the involvement of Ni in the NH_4_^+^ storage process. Figure 4b,c depict the Ni 2*p* XPS spectra at charged and discharged states, respectively. In the charged state (Figure 4b), the peaks at binding energies of 856.2 and 873.8 eV are assigned to Ni^2+^, while those at 857.5 and 875.48 eV are ascribed to Ni^3+^. The integral area ratio of Ni^2+^ to Ni^3+^ is 1.17, giving an average Ni valence state of +2.46.^[^ ^47^, ^48^ ^]^ Furthermore, in the discharged state (Figure 4c), the peaks at binding energy of 856.3 and 874.0 eV correspond to Ni^2+^, and peaks at 858.2 and 876.5 eV indicate Ni^3+^, with an integral area ratio of 1.87, suggesting an average valence state of +2.35.^[^ ^47^, ^48^ ^]^ The transition from +2.46 to +2.35 between charged and discharged states demonstrates partial electron transfer during cycling, actively participating in the redox reactions associated with NH_4_^+^ storage. In the NiHCF system, Ni plays a dual role during NH_4_^+^ storage, including directly providing electrons for NH_4_^+^ storage and engaging in electronic interactions with Fe. The electrons involved in the Ni─Fe interactions also indirectly participate in the NH_4_^+^ storage process. The extent of Ni's contribution to NH_4_^+^ storage can be quantified through valence state variations of Ni. XANES and XPS analyses reveal that the average valence state of Ni shifts between +2.46 and +2.35 during charge/discharge cycles, accounting for approximately 24% of the total NH_4_^+^ storage capacity. In the NiN_6_ octahedron, the outermost electron configuration of Ni changes from 3*d*_8_4*s*_2_ to 3*d*_7_4*s*_2_ as Ni^2+^ loses an electron, inducing Jahn‐Teller distortion. However, the partial electron transfer minimizes structural distortion, maintaining the integrity of the framework. This behavior underscores the significance of ligand field modulation in activating Ni as a redox center without compromising structural stability. Similarly, the Fe K‐edge XANES spectra and Fe 2*p* XPS spectra provide insights into the role of Fe during cycling in AIBs. According to K‐edge XANES spectra (Figure S25, Supporting Information), the shift of Fe K‐edge to lower energy in the charged state than that in a discharged state demonstrates that Fe participates in NH_4_^+^ storage. In Fe 2*p* XPS spectra (Figure 4d,e), the peaks at binding energies of 708.2 and 721.1 eV are assigned to Fe^2+^, and those at 709.9 and 723.3 eV are attributed to Fe^3+^ in the charged state.^[^ ^39^ ^]^ The integral area ratio of Fe^2+^ to Fe^3+^ is 0.85, indicating an average Fe valence state of +2.54. In the discharged state, the binding energies of 708.5 and 721.4 eV are assigned to Fe^2+^, giving an average valence state of +2.00.^[^ ^39^ ^]^ The incomplete transition of Fe to +3 in the charged state suggests that partial NH_4_^+^ ions act as structural pillars to support the framework, with Fe providing charge compensation to stabilize these NH_4_^+^ ions.^[^ ^55^ ^]^ These observations confirm that while both Ni and Fe participate in the redox reactions, Fe contributes more significantly to the electron transfer involved in NH_4_^+^ ion storage.

To further probe the structural variations of NiHCF during electrochemical cycling, the extended X‐ray absorption fine structure (EXAFS) analyses are performed (Figure 4f). The R‐space EXAFS curves reveal changes in atomic distances within NiHCF at different states. At a pristine state, the main peaks at 1.45, 2.44, and 4.74 Å are attributed to atomic distances of Ni─N, Ni─C, and Ni─Fe, respectively.^[^ ^56^ ^]^ At charged (0.8 V) and discharged (0 V) states, the Ni─N and Ni─C peaks exhibit notable peak splitting. Specifically, the Ni─N peak at 1.45 Å splits into peaks at 1.09 and 1.61 Å in the charged state, with similar splitting observed in the discharged state. Similarly, the Ni─C peak at 2.44 Å shifts to 2.15 and 2.61 Å in both charged and discharged states. This peak splitting indicates structural distortions resulting from the interaction between NH_4_^+^ ions and the N atoms in NiHCF, leading to elongation or contraction of bonds within the Ni─N≡C─Fe linkage.^[^ ^40^ ^]^ According to Fe R‐space EXAFS spectra (Figure S26, Supporting Information), the atomic distances of Fe─C and Fe─N in the discharged state shift to the lower distances compared to that in charged state, attributing to structural distortions after NH_4_^+^ intercalating. Wavelet transform EXAFS analyses provide a detailed examination of local structural changes by highlighting the specific atomic distances associated with Ni─N, Ni─C, and Ni─Fe shells (Figure 4g–i).^[^ ^57^ ^]^ Unlike the R‐space EXAFS spectra, the wavelet transform does not show peak splitting but reveals variations in the intensity and position of the sub‐lobes corresponding to different atomic interactions. Notably, the Ni─N bond length in the discharged state is slightly longer than in the pristine and charged states (Figure S27, Supporting Information), suggesting bond elongation upon NH₄⁺ intercalation. In contrast, the atomic distances of Ni─C and Ni─Fe show minimal changes across different states. Furthermore, the Fe─C bond length in the discharged state is slightly lower than in the pristine and charged states (Figure S28, Supporting Information). These EXAFS results suggest that the Ni─N≡C─Fe chain undergoes reversible structural distortions during electrochemical cycling, primarily involving the Ni─N bonds affected by NH₄⁺ interaction. The partial electron transfer and resulting ligand field modulation facilitate these structural adjustments without causing significant framework degradation. The ability of NiHCF to accommodate these changes contributes to its superior cycling performance and structural stability.

**Figure 5:**
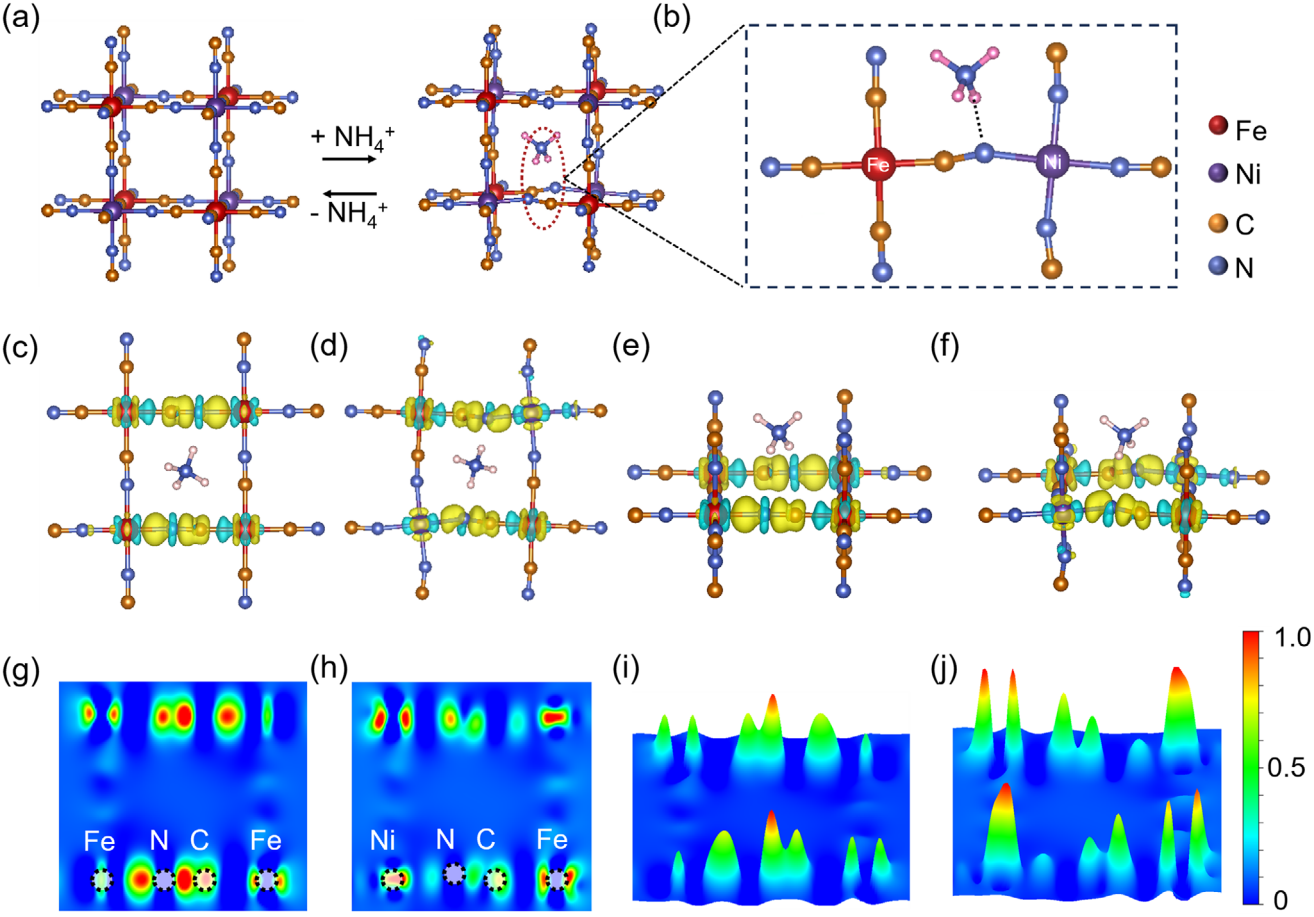
The schematic diagrams and charge density distribution in NH_4_^+^ storage processes for FeHCF and NiHCF. a) Schematic representation of the NH_4_^+^ ion storage process in NiHCF. b) Local zoom image of NiHCF after NH_4_^+^ ion intercalating. c,d) The front view patterns of different charge density distributions for FeHCF and NiHCF. e,f) The side view patterns of different charge density distributions for FeHCF and NiHCF. The yellow area represents charge accumulation, and the cyan area represents charge depletion. g,h) 2D contour plots of electron localization function for FeHCF and NiHCF. i,j) 3D contour plots of electron localization function for FeHCF and NiHCF.

The enhancement of the redox potential in NiHCF is further elucidated through detailed analyses and computational studies. As previously confirmed by XANES and XPS analyses, electron transfer from Ni^2+^ to Fe^3+^, primarily facilitated by electron rearrangement within the crystal field. This electron transfer not only contributes to the stability of the NiHCF framework but also plays a pivotal role in elevating the redox potential. Previous reports have identified N atoms in PBAs as the principal NH_4_^+^ storage sites in AIBs, with the absorption energy between N atoms and NH_4_^+^ critically influencing operational potential.^[^ ^31^, ^36^, ^58^ ^]^ Herein, density functional theory (DFT) calculations were employed to quantify the absorption energy between N and NH_4_^+^ in NiHCF and FeHCF. As shown in Table S8 (Supporting Information), the calculated absorption energy for NH_4_^+^ on the N site in NiHCF is −5.26 eV, which is more negative than that in FeHCF (−4.80 eV). This indicates a stronger interaction in NiHCF, correlating with a higher NH_4_^+^ storage potential.^[^ ^58^ ^]^ These findings align well with the CV curves and galvanostatic charging and discharging results, demonstrating a higher redox potential for NiHCF compared to FeHCF. The enhanced absorption energy between the N atom and NH_4_^+^ leads to the notable shift in N toward NH_4_^+^ and induces structural distortions within the Ni─N≡C─Fe linkage (**Figure** **5**a,b), as evidenced by our structural analyses. This structural adjustment is facilitated by the ligand field‐induced electron transfer, which modifies the electronic environment and enhances the interaction between NH_4_^+^ and the framework.

To further elucidate the role of Ni substitution in charge transfer mechanisms, differential charge density analyses were conducted. Figure 5c–f highlights distinct patterns of charge accumulation and depletion in NiHCF and FeHCF upon NH_4_^+^ interaction. In the FeHCF framework (Figure 5c,e), charge accumulation is observed along the Fe─N bond, C≡N bond, and C atom, whereas charge depletion primarily occurs in Fe atoms interacting with N and C atoms. In comparison, for NiHCF (Figure 5d,f), a more pronounced charge depletion is observed around Ni atoms, and charge accumulation is observed around Fe atoms, with the accumulation pattern changing in response to the distortion of the Ni─N≡C─Fe bond. This highlights electron transfer along this bond and underscores the significance of Ni substitution in modulating the electronic structure. Contour plots of electron localization function (ELF) were utilized to dissect the electron distribution within the NiHCF and FeHCF frameworks (Figure 5g–j). The ELF values ranging 0.5–1.0 indicate regions with localized bonding and non‐bonding electrons, while values between 0 and 0.5 suggest delocalized electrons.^[^ ^59^, ^60^ ^]^ In NiHCF, a pronounced concentration of localized electrons around Ni and Fe atoms is observed (Figure 5h,j), contrasting with FeHCF, where electron localization is primarily around the Fe─N and N≡C bonds (Figure 5g,i). This redistribution of electrons in NiHCF underscores the substantial role of Ni substitution in enhancing NH_4_^+^ absorption, thereby contributing to the higher operational potential of NiHCF in AIBs.

**Figure 6:**
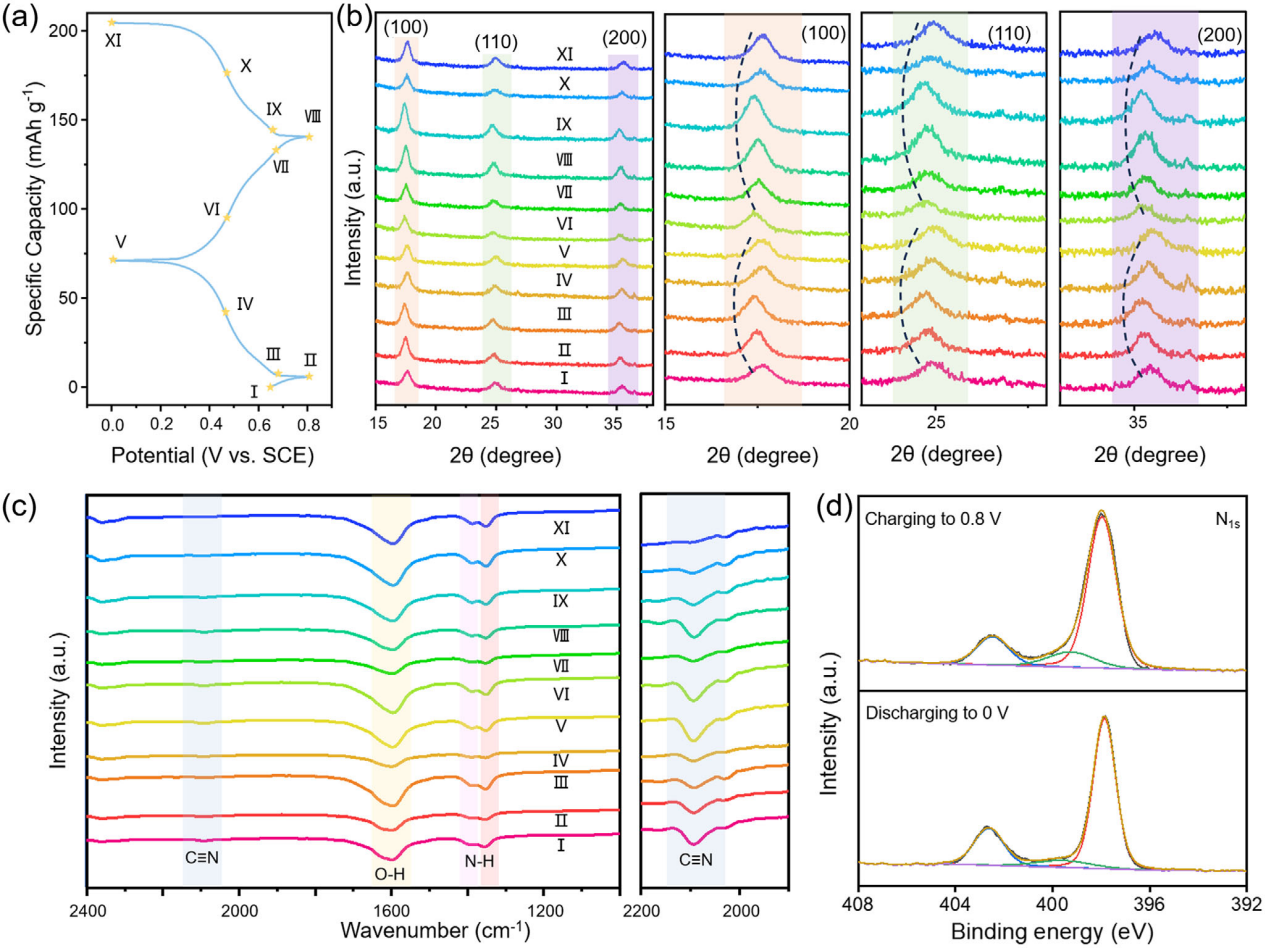
Ex situ characterizations of NiHCF at different charge–discharge states in AIBs. a) Galvanostatic charging and discharging profiles. b) Ex situ XRD patterns illustrating lattice spacing variations corresponding to the intercalation and de‐intercalation of NH_4_^+^ ions. c) Ex situ FTIR spectra, highlighting the N─H bending vibrations associated with NH_4_^+^ hydrogen bonding and structural interactions within NiHCF. d) N1*s* XPS spectra showing peaks for C≡N, N─H, and N⁺─H, confirming NH_4_^+^ adsorption and intercalation into the NiHCF framework at various charge–discharge states.

The reversible structure variations and bonding information of NiHCF during cycling were investigated by ex situ XRD, FTIR, and XPS analyses, as shown in **Figure** **6**. The ex situ XRD patterns reveal the variations in lattice spacing during the first two galvanostatic charging and discharging cycles within a potential window of 0–0.8 V (vs SCE). In Figure 6a,b, the characteristic peaks of NiHCF corresponding to the crystal planes of (100), (110), (200) shift to lower 2θ values during the charging process, demonstrating the lattice expansion due to NH_4_^+^ de‐intercalation. The detailed lattice spacings at charged and discharged states have been shown in Table S9 (Supporting Information). Conversely, during the discharging process, these peaks shift to higher 2θ values, signifying that NH_4_^+^ intercalates back into NiHCF. This pattern indicates that the electrostatic adsorption between NH_4_^+^ and NiHCF framework is stronger than electrostatic repulsion, which is different from the behavior observed in PBAs in other ion batteries.^[^ ^61^ ^]^ Furthermore, peak intensities increase during the charging process and decrease during the discharging process (Figure S29, Supporting Information), suggesting reduced crystallinity due to structural distortion when NH_4_^+^ intercalates. These findings are consistent with EXAFS and DFT analyses and demonstrate the highly reversible nature of NH_4_^+^ intercalation and de‐intercalation in NiHCF. Detailed bonding information was further obtained from ex situ FTIR spectra (Figure 6c). The characteristic peaks at 2100 and 1600 cm^−1^ correspond to the stretching vibration of C≡N and ─OH groups, respectively.^[^ ^62^ ^]^ Two additional peaks at 1349 and 1393 cm^−1^ correspond to the bending vibrations of N─H bonds. The former peak indicates hydrogen bonding between H of NH_4_^+^ and N of NiHCF, while the latter peak can be ascribed to the N─H of NH_4_^+^.^[^ ^36^ ^]^ These FTIR results demonstrate the existence of hydrogen bonding during charging and discharging cycles, which is consistent with previous reports.^[^ ^32^, ^36^ ^]^ The NH_4_^+^ ions storage mechanism is further corroborated by XPS N1*s* fine spectra. The characteristic peaks at 397.9, 399.5, and 402.7 eV correspond to C≡N, N─H, and N^+^─H, respectively (Figure 6d).^[^ ^63^ ^]^ The existence of N^+^─H manifests the adsorbed and intercalated NH_4_^+^ ions. Compared with the charge to 0.8 V (vs SCE), the content ratio of N^+^─H in NiHCF increases during discharge to 0 V (vs SCE), further confirming the intercalation of NH_4_^+^ into the NiHCF framework. These comprehensive analyses confirm that Ni substitution enhances the redox potential and operational performance of NiHCF in AIBs through a ligand field‐induced synergistic dual active sites mechanism. The electron transfer from Ni to Fe, facilitated by ligand field modulation, activates Ni as an additional redox center and strengthens the interaction between NH_4_^+^ and the N atoms in the framework. This leads to higher NH_4_^+^ adsorption energy, elevated redox potential, and improved structural stability during cycling.

**Figure 7:**
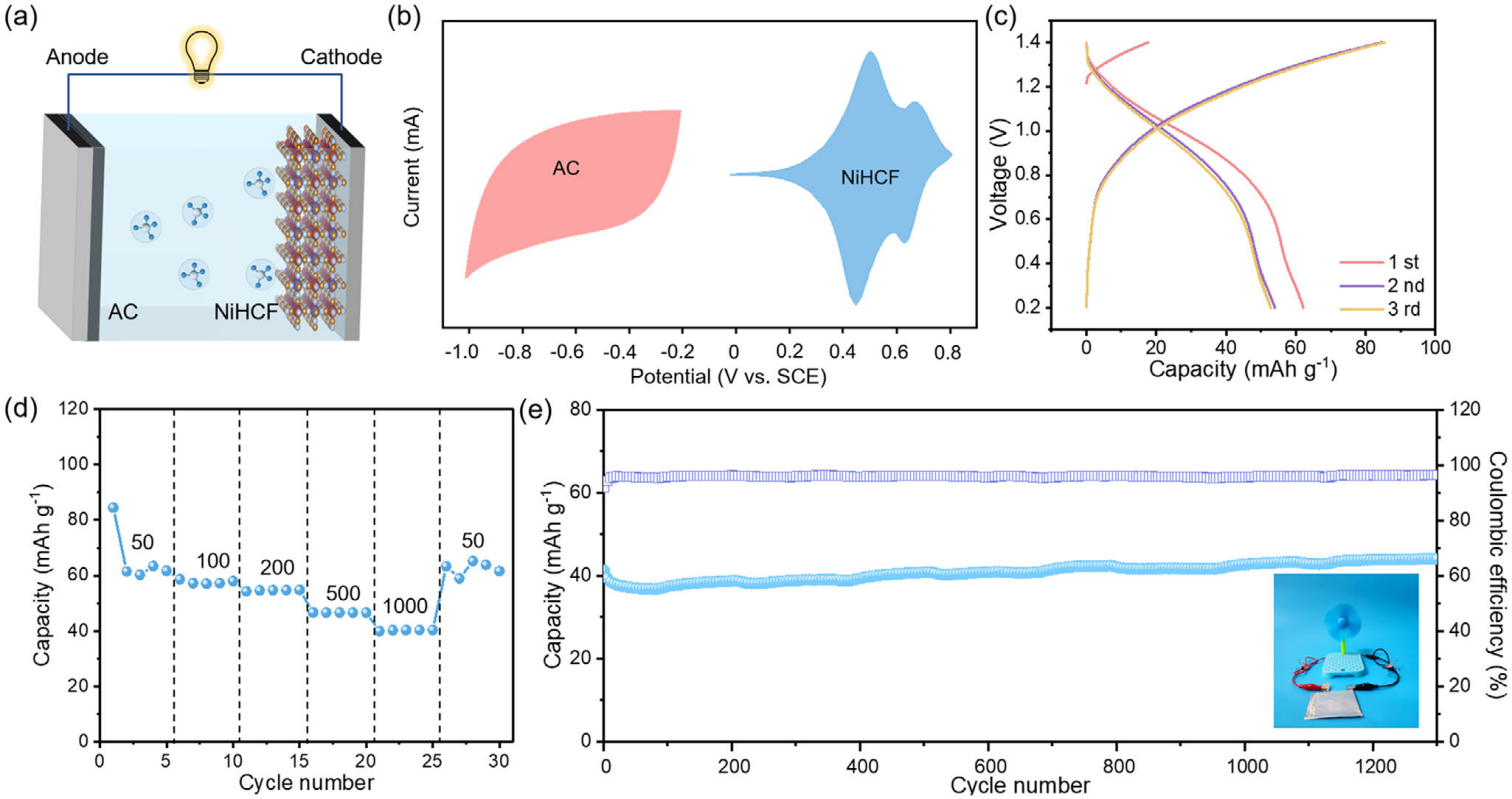
The electrochemical performance of NiHCF//AC full cell. a) Schematic of ammonium ion full cell based on AC as anode, NiHCF as cathode, and 2 m ammonium acetate as electrolyte. b) The working potential matchup between AC and NiHCF. c) The first three galvanostatic charging and discharging profiles of NiHCF//AC full cell at a current density of 50 mA g^−1^. d) The rate performance of NiHCF//AC full cell. e) The cycling performance of NiHCF//AC full cell.

An ammonium‐ion full cell is assembled with NiHCF as the cathode, activated carbon (AC) as the anode, and 2 m ammonium acetate as the electrolyte (**Figure** **7**a). As shown in Figure 7b, the AC operates within a potential window of −1.0 to −0.2 V (vs SCE), while the NiHCF cathode functions between 0 and 0.8 V (vs SCE). The NiHCF//AC full cell delivers a discharge capacity of 62.2 mAh g^−1^ and an energy density of 56.0 Wh kg^−1^ under an operating voltage range of 0.2–1.4 V at 50 mA g^−1^ (Figure 7c). Furthermore, the cell demonstrates exceptional rate capability (Figure 7d), achieving discharge capacities of 61.5, 58.6, 54.3, 46.7, and 39.9 mAh g^−1^ at current densities of 50, 100, 200, 500, and 1000 mA g^−1^, respectively. Remarkably, the capacity recovers to 59.0 mAh g^−1^ when the current density is reset to 50 mA g^−1^, highlighting excellent rate performance. Additionally, the NiHCF//AC full cell exhibits ≈100% capacity retention after 1400 cycles at 1000 mA g^−1^, indicating ultrahigh cycling stability (Figure 7e). The gradual increase in discharge capacity during cycling may be attributed to progressive electrolyte infiltration into the NiHCF electrode, which enables the sequential activation of previously inaccessible electrochemically active sites.^[^ ^64^, ^65^, ^66^, ^67^ ^]^ Compared to other reported full cells (Table S10, Supporting Information), the NiHCF//AC system demonstrates superior electrochemical performance and exceptional application potential. To further validate its practical applicability, a prototype pouch cell was assembled and successfully powered a small fan, underscoring the promising energy storage value and potential for real‐world implementation.

## 3 Conclusion

In summary, we have systematically investigated the implications of Ni substitution in nickel hexacyanoferrate (NiHCF) for NH_4_^+^ ion storage in aqueous ammonium ion batteries (AIBs). Through advanced characterization techniques and computational studies, we demonstrated that Ni substitution induces ligand field‐induced electron transfer from Ni^2^⁺ to Fe^3^⁺ within the Ni─N≡C─Fe chain, activating Ni as an additional redox center. This ligand field‐induced dual active sites mechanism enhances the redox potential and overall electrochemical performance of NiHCF. NiHCF exhibits higher redox potentials of 0.45/0.51 V and 0.63/0.67 V (vs SCE) and achieves a capacity of 61.3 mAh g^−1^ at 50 mA g^−1^, superior rate performance, and robust cycling stability with 71.1% capacity retention after 1000 cycles. The enhanced performance is attributed to the synergistic effects of the dual active sites and the optimized charge distribution within the framework resulting from the ligand field‐induced electron transfer. Ex situ characterizations confirm the reversible structural variations during charging and discharging processes, supporting the proposed mechanism. Our findings provide a comprehensive understanding of how Ni substitution influences the electronic structure and operating mechanism of PBAs in AIBs. By revealing the higher adsorption energy between NH₄⁺ and N atoms in NiHCF, we have elucidated the structural and electronic factors contributing to the enhanced performance. This work offers pivotal guidance for future research on PBAs and opens new avenues for designing high‐energy density cathode materials for AIBs. Further exploration of transition metal substitution and ligand field modulation in PBAs may lead to the development of advanced electrode materials for next‐generation energy storage systems.

## Conflict of Interest

The authors declare no conflict of interest.

## Supporting information



Supporting Information

## Data Availability

The data that support the findings of this study are available from the corresponding author upon reasonable request.
