# Supplementary material for: Ligand Field‐Induced Dual Active Sites Enhance Redox Potential of Nickel Hexacyanoferrate for Ammonium Ion Storage
Source: Adv Mater. 2025 May 20;37(32):2419446. doi: 10.1002/adma.202419446 (PMC12355446; doi:10.1002/adma.202419446)
Supplement: Supplementary file 1 — Supporting Information [file ADMA-37-2419446-s001.docx]

Supporting Information

**Ligand Field-Induced Dual Active Sites Enhance Redox Potential of Nickel Hexacyanoferrate for Ammonium Ion Storage**

*Mengmeng Zhou^a,e^, Tong Wu^b^,* *Mengde Kang^c^, Tengfei Cheng^d^, Hui Li^e^, Liqing He^d^*, Cheng Lian^c^, Tianyi Ma^e^*, Qin Zhao^a^**

^a^M. Zhou, Dr. Q. Zhao
Key Laboratory for Green Synthesis and Preparative Chemistry of Advanced Materials of Liaoning Province, Institute of Clean Energy Chemistry College of Chemistry
Liaoning University
Shenyang, 110036, China
E-mail: [zhaoqin@lnu.edu.cn](mailto:zhaoqin@lnu.edu.cn)

^b^Dr. T. Wu
Inner Mongolia Engineering Research Centre of Lithium-Sulfur Battery Energy Storage

College of Chemistry and Materials Science

Inner Mongolia Minzu University

Tongliao, 028000, China

^c^M. Kang, Prof. C. Lian

School of Chemistry and Molecular Engineering

East China University of Science and Technology

Shanghai, 200237, China

^d^T. Cheng, Dr. L. He
Hefei General Machinery Research Institute Co., Ltd

Hefei, 230031, China

E-mail: [heli_limao@163.com](mailto:heli_limao@163.com)

^e^M. Zhou, Dr. H. Li, Prof. T. Ma
Centre for Atomaterials and Nanomanufacturing (CAN)
School of Science, RMIT University
Melbourne, VIC 3000, Australia
E-mail: [tianyi.ma@rmit.edu.au](mailto:tianyi.ma@rmit.edu.au)

1. **Materials preparation**

**NiHCF Synthesis**. 1 mmol NiCl_2_•6H_2_O was dissolved in 25 ml 0.2 M HCl to form solution A. In a separate container, 1 mmol K_3_Fe(CN)_6_ was dissolved in 25 ml 0.2 M HCl to obtain solution B. Solution A was slowly added dropwise to solution B with continuous stirring, resulting in the formation of tawny precipitates. The mixed solution was stirred for 12 hours, and the precipitates were subsequently obtained by centrifugation and washed by deionized water. The obtained NiHCF powder was dried in vacuum oven at 60 ºC for 12 hours.

**FeHCF Synthesis**. 1 mmol FeCl_2_•2H_2_O was dissolved in 25 ml 0.2 M HCl to obtain solution A. 1 mmol K_3_Fe(CN)_6_ was dissolved in 25 ml 0.2 M HCl to obtain solution B. Solution A was added dropwise to solution B with continuous stirring, resulting in the formation of blue precipitate. The mixed solution should be stirred for 12 h and the precipitates can be obtained by centrifugation and washed by deionized water. The obtained FeHCF powder was dried in vacuum oven at 60 ºC for 12 h.

1. **Material Characteristics**

Scanning electron microscopy (SEM) images were collected by a SU8010-type field emission scanning electron microscope. Energy dispersive spectrometer (EDS) mapping images were collected using an APOLLO.XL-type X-ray dispersive spectrometer. Transmission electron microscopy (TEM) images were collected using JEM 2100 microscopy. X-ray diffraction (XRD) patterns for NiHCF and FeHCF, and the ex-situ XRD of working electrodes, were recorded on a Bruker D8-Advance powder X-ray diffractometer (Cu K*α*). The functional groups of NiHCF and FeHCF were characterized by Raman spectroscopy using a LabRAM HR Evolution-HORIBA system. Thermogravimetry analysis (TGA) curves were tested by SDT Q600-type simultaneous thermal analyzer from room temperature to 1000 ℃ and the rate of temperature increase is 5 ºC min^-1^. The elements contents for NiHCF and FeHCF were confirmed by: Aglient 5110 inductively coupled plasma Optical Emission Spectrometer (ICP-OES). X-Ray absorption fine spectroscopy (XAFS) was performed using a Table XAFS-500 (Specreation Instruments Co., Ltd). X-ray photoelectron spectroscopy (XPS) was performed by Thermo Kalpha spectroscopy. Ex-situ Fourier Transform Infrared Spectroscopy patterns (FTIR) were achieved by NICOLET6700 Fourier transform infrared absorption spectrometer.

1. **Electrochemical Measurements**

Ammonium ion batteries (AIBs) were assembled with NiHCF or FeHCF as the working electrode, a carbon rod as the counter electrode, a saturated calomel electrode as the reference electrode and a 2 M ammonium acetate solution as the electrolyte. Working electrode was prepared by evenly spreading the slurry on Ti foils, followed by drying at 60 ºC for 12 h in a vacuum oven. The slurry was composed of 70 wt% active materials (NiHCF or FeHCF), 20 wt% acetylene black, and 10 wt% of polyvinylidene fluoride (PVDF) with blending with N-methylpyrone (NMP). The mass loading of active materials on the working electrodes was approcimately 1-2 mg cm^-2^.

1. **Kinetic process equations**

$D=\frac{4}{\pi\tau}{(\frac{n_{M}V_{M}}{S})}^{2}({\frac{\Delta E_{S}}{\Delta E_{\tau}})}^{2}$,^[1]^ (1)

*i* = *av^b^*,^[2]^ (2)

*i*(*V*) = *k*_1_*v* + *k*_2_*v*^1/2^,^[3]^ (3)

In **equation 1,** τ, n_M_, V_M_, S, ΔE_S_, and ΔE_τ_ represent pulse time (s), molar number (mol), molar volume (cm^3^ mol^-1^), contact area (cm^2^), voltage variation caused by galvanostatic charge discharge process, and the voltage variation due to the current pulse. In **equation 2**, the values of “*a*” and “*b*” are constants. The “*b*” value of 0.5 manifests that the NH_4_^+^ storage process is predominantly controlled by diffusive behavior, whereas the “*b*” value of 1 indicates the NH_4_^+^ storage process controlled by capacitive behavior. In **equation 3**, *k*_1_*v* and *k*_2_*v*^1/2^ indicate capacitive and diffusive contributions, respectively.

1. **Computational Details.**

The absorption energy for NH_4_^+^ on NiHCF was calculated using density functional theory (DFT) with the projector augmented plane-wave method. The generalized gradient approximation proposed by Perdew-Burke-Ernzerhof (PBE) is selected for the exchange-correlation potential. The cut-off energy for plane wave is set to 480 eV. The energy criterion is set to 10^−5^ eV in the iterative solution of the Kohn-Sham equation. All the structures are relaxed until the residual forces on the atoms have declined to less than 0.02 eV/Å.

The adsorption energy Eads is expressed as:

E_absorption_ = E_complex_ – E_slab_ – E_ammonium_ (4)

In **equation (4)**, E_complex_ is the total energy of slab Prussian blue model with NH_4_^+^ adsorption, E_slab_ is the energy of a slab Prussian blue, and $E_{B}$ is that for a NH_4_^+^. The detailed energy is shown in **Table S4**.

The calculations of difference charge density are performed in the framework of the DFT with the projector augmented plane-wave method. The generalized gradient approximation proposed by Perdew-Burke-Ernzerhof (PBE) is selected for the exchange-correlation potential. The cut-off energy for plane wave is set to 480 eV. The energy criterion is set to 10^−4^ eV in the iterative solution of the Kohn-Sham equation. All the structures are relaxed until the residual forces on the atoms have declined to less than 0.05 eV/Å. To avoid interlaminar interactions, a vacuum spacing of 20 Å is applied perpendicular to the slab. Here, we define **equation (5)** as the charge density difference of A/B heterostructure.

Δρ = ρ_complex_ – ρ_slab_ – ρ_ammonium_ (5)

In **equation (5)**, ρ_complex_ , ρ_slab_ and ρ_ammonium_ are the charge densities of NH_4_^+^-Prussian blue heterostructure, isolated slab Prussian blue and slabs NH_4_^+^, respectively.


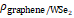

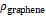


1. **Supplemental Figures**


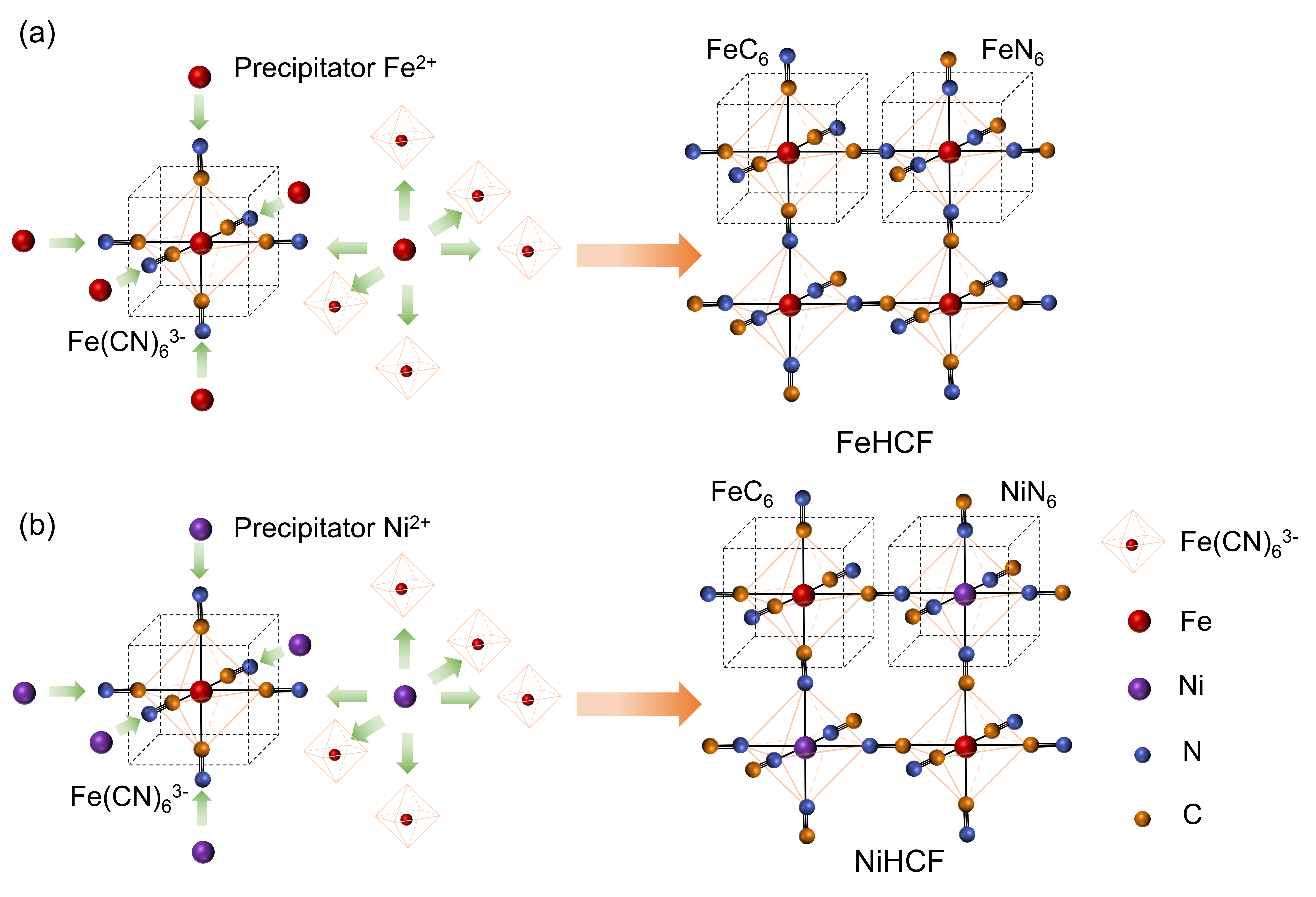


**Figure S1** The synthesis schematic of (a) FeHCF and (b) NiHCF.

The synthesis of FeHCF involves the coordination of Fe^2+^ with the N atoms of [Fe(CN)_6_]^3-^, forming a three-dimensional (3D) open framework composed of alternating FeC_6_ (C-coordinated Fe^3+^) and FeN_6_ (N-coordinated Fe^2+^) octahedra (**Figure S1a**). In contrast, NiHCF is synthesized by replacing Fe^2+^ with Ni^2+^ as the precipitator ion. During this process, Ni^2+^ selectively binds to the N atoms of [Fe(CN)_6_]^3-^, yielding a structurally analogous 3D framework with alternating FeC_6_ (C-coordinated Fe^3+^) and NiN_6_ (N-coordinated Ni^2+^) octahedra (**Figure S1b**). Critically, the substitution of Fe^2+^ by Ni^2+^ occurs exclusively at the N-coordinated sites, while the C-coordinated Fe^3+^ units remain intact.


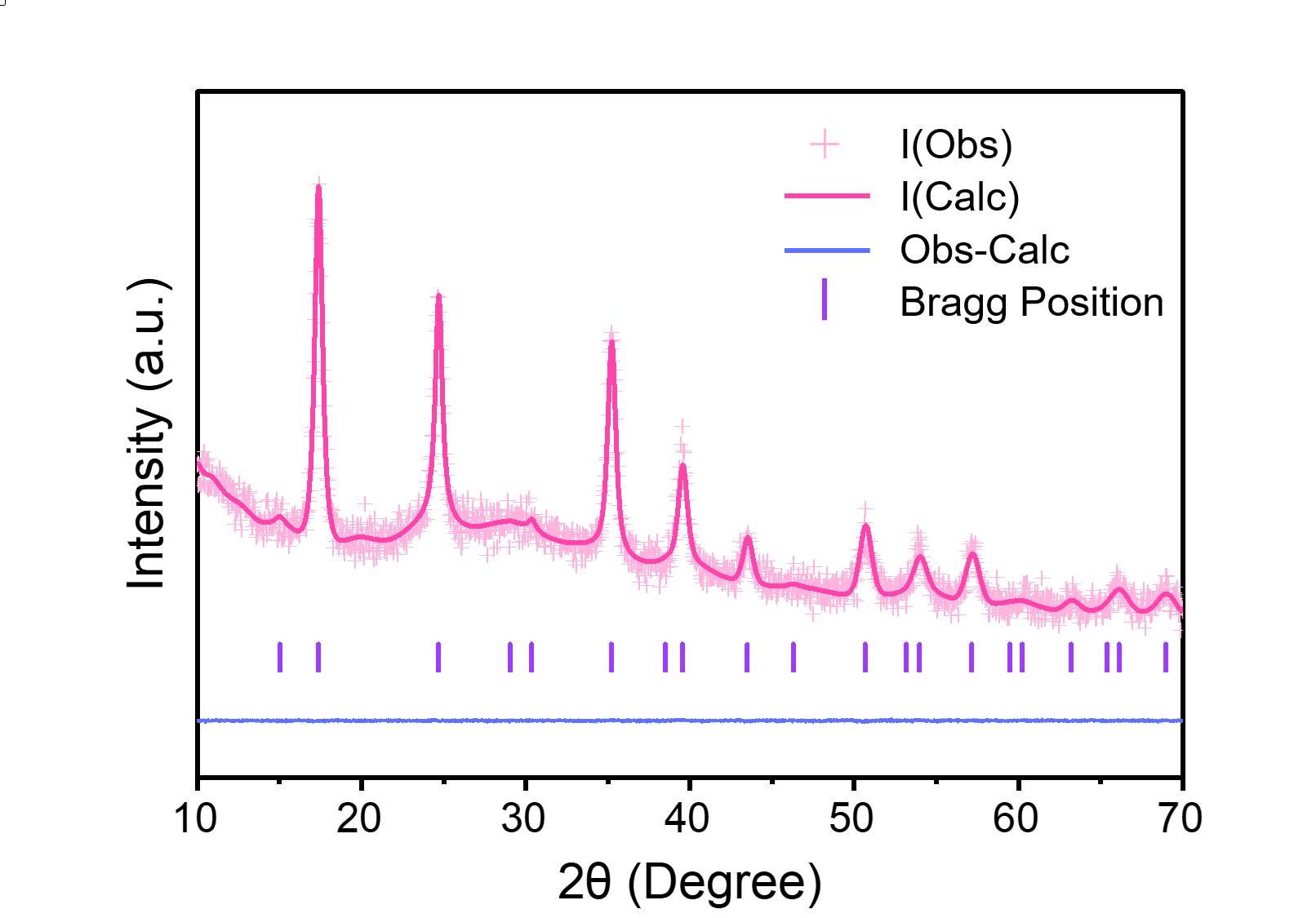


**Figure S2** The XRD pattern and Rietveld refinement of FeHCF (R_wp_=5.798%).

**
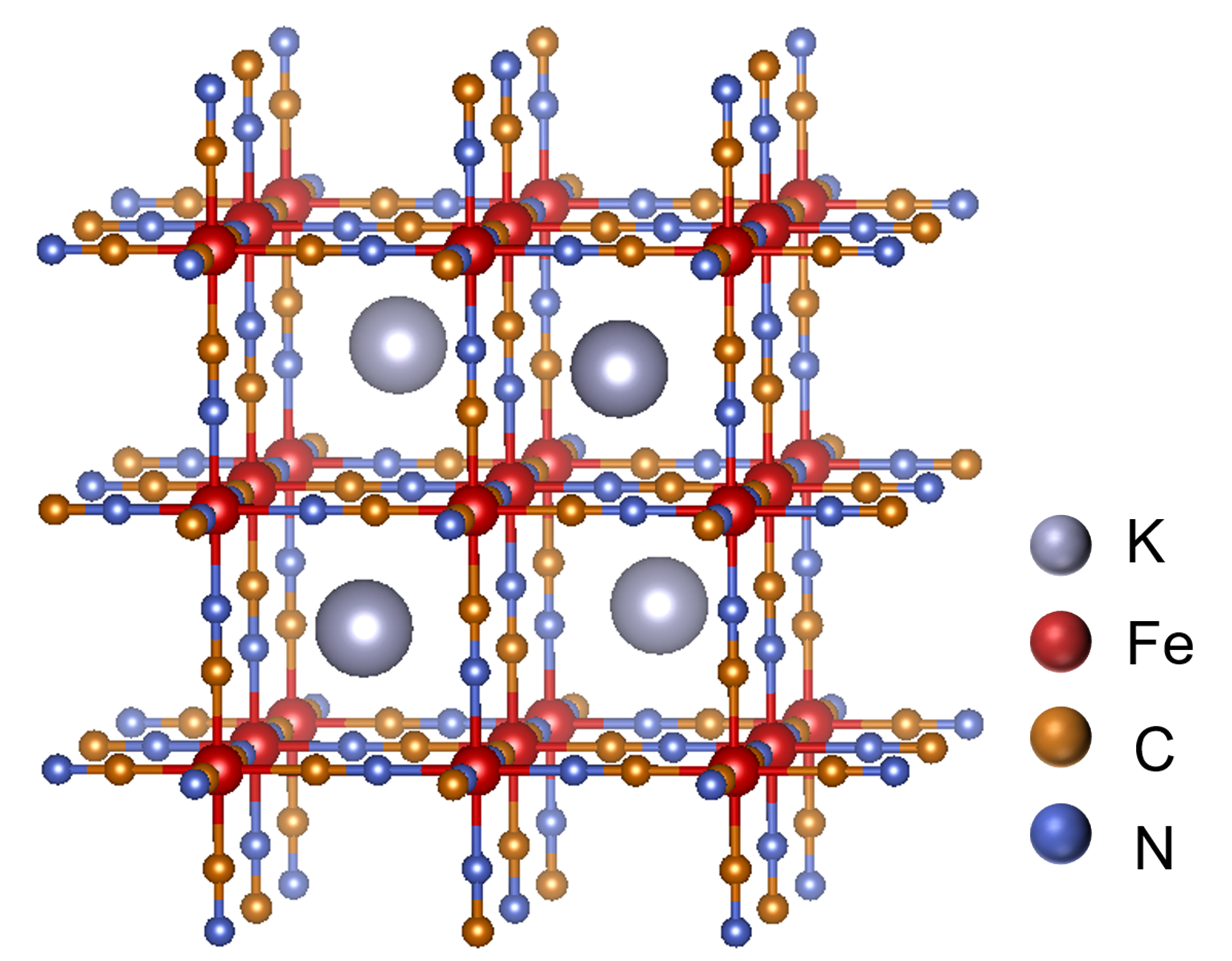
**

**Figure S3** Structure diagram of FeHCF. The FeHCF framework is constructed by FeC_6_ octahedrons with low spin state and FeN_6_ octahedrons with high spin state.


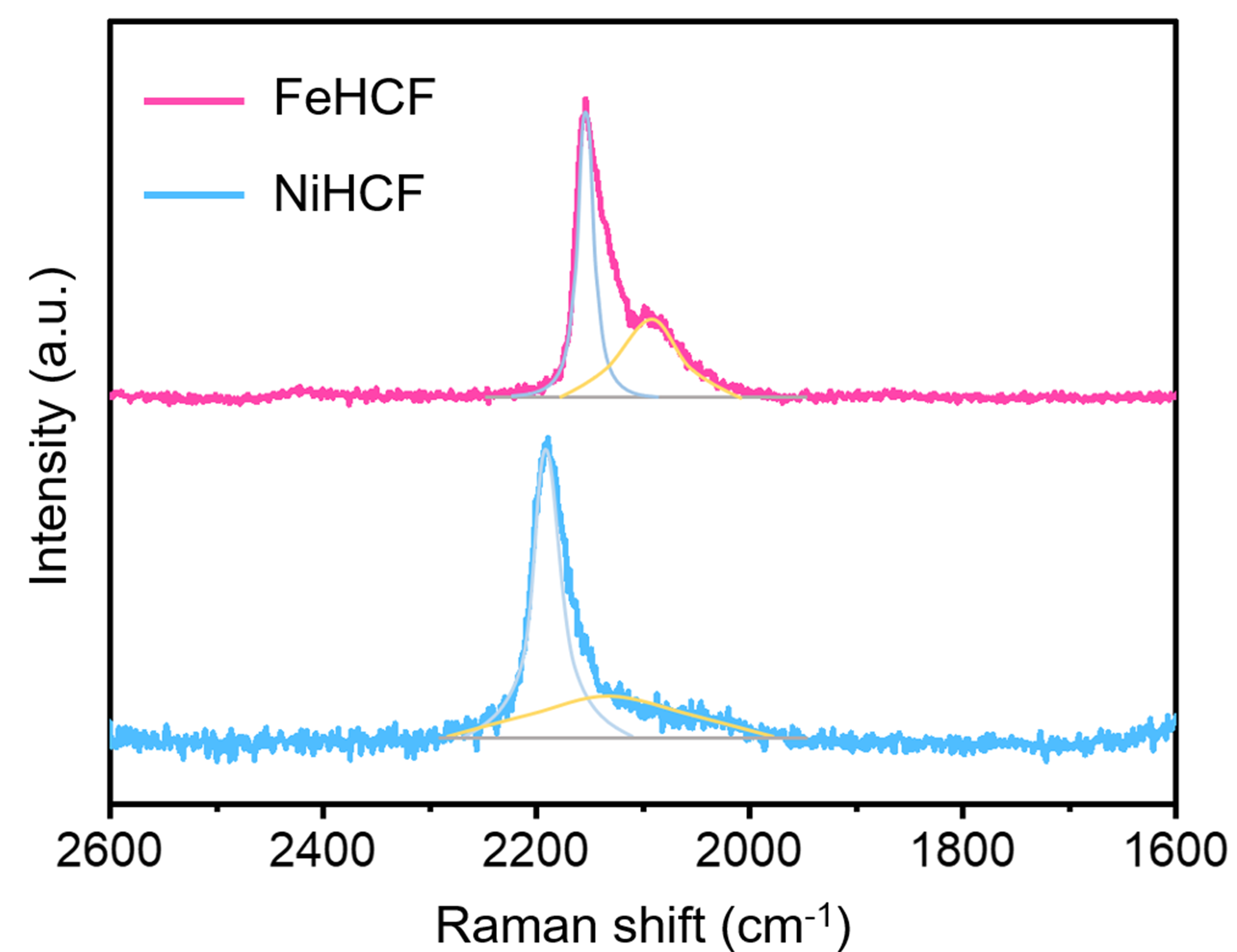


**Figure S4** The Raman spectra of NiHCF and FeHCF. In NiHCF, the characteristic peaks at 2192 cm^-1^ and 2132 cm^-1^ are assigned to the C≡N stretching vibrations of Fe-C≡N-Ni, while FeHCF displays characteristic peaks at 2154 cm^-1^ and 2095 cm^-1^ corresponding to the C≡N stretching vibrations of Fe-C≡N-Fe vibrations.^[4, 5]^


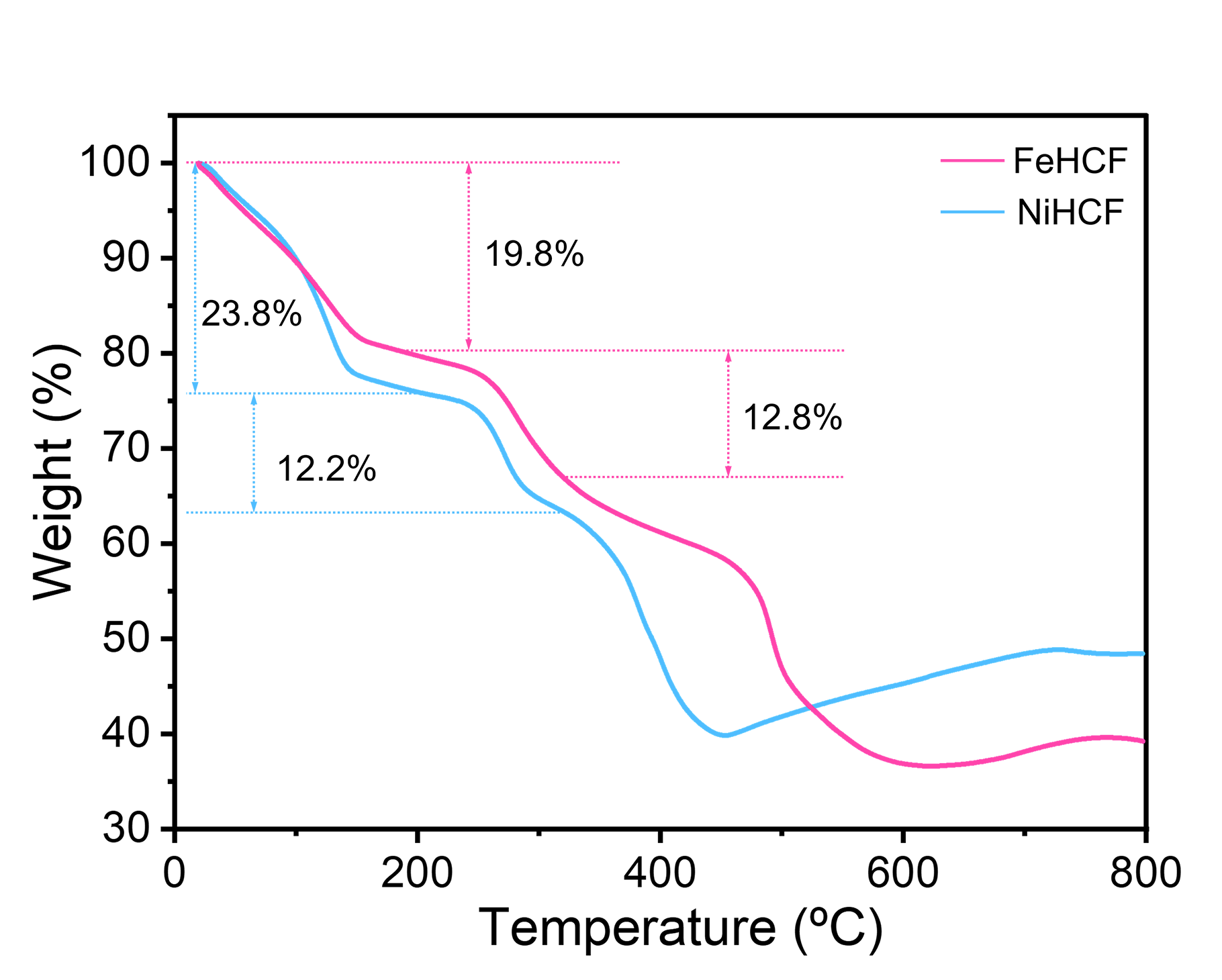


**Figure S5** The TGA curves of NiHCF and FeHCF. The weight losses below 200 ºC can be due to absorbed water and between 200-300 ºC from crystal water.^[6]^ According to the results of TGA, NiHCF and FeHCF have similar crystal water contents of 12.2% and 12.8%. The higher content of crystal water represents many defects in Prussian blue and its analogues, which can promote the NH_4_^+^ storage in aqueous AIBs.


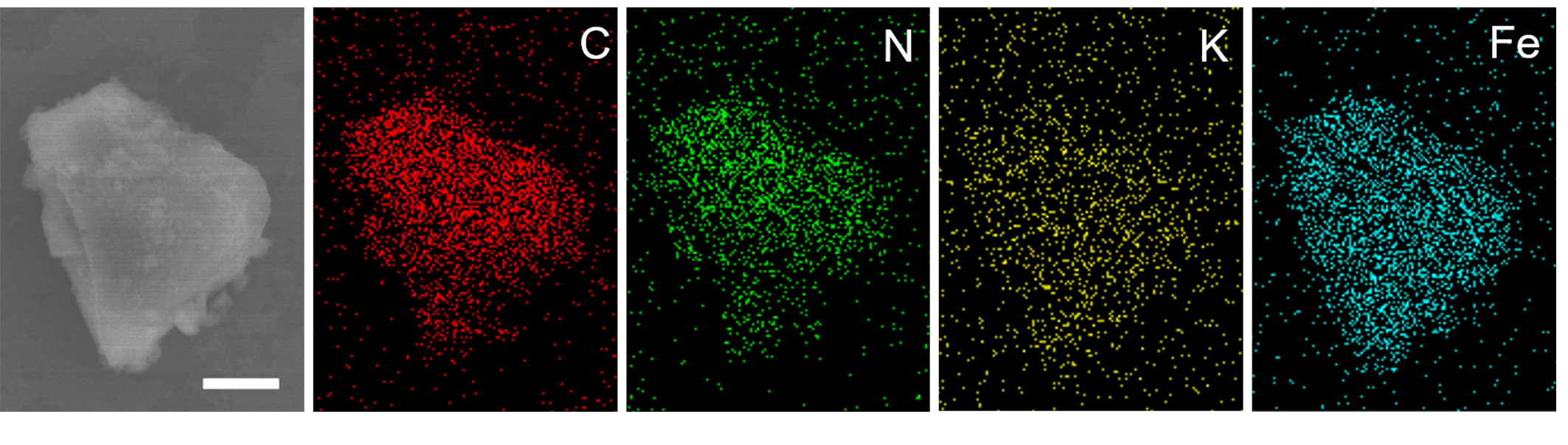


**Figure S6** SEM image and its EDS mapping of element distributions for FeHCF with the scale plate of 3 μm. SEM image reveals that FeHCF shows the massive morphology. The EDS mapping images display the uniform distributions of C, N, K, Fe elements.


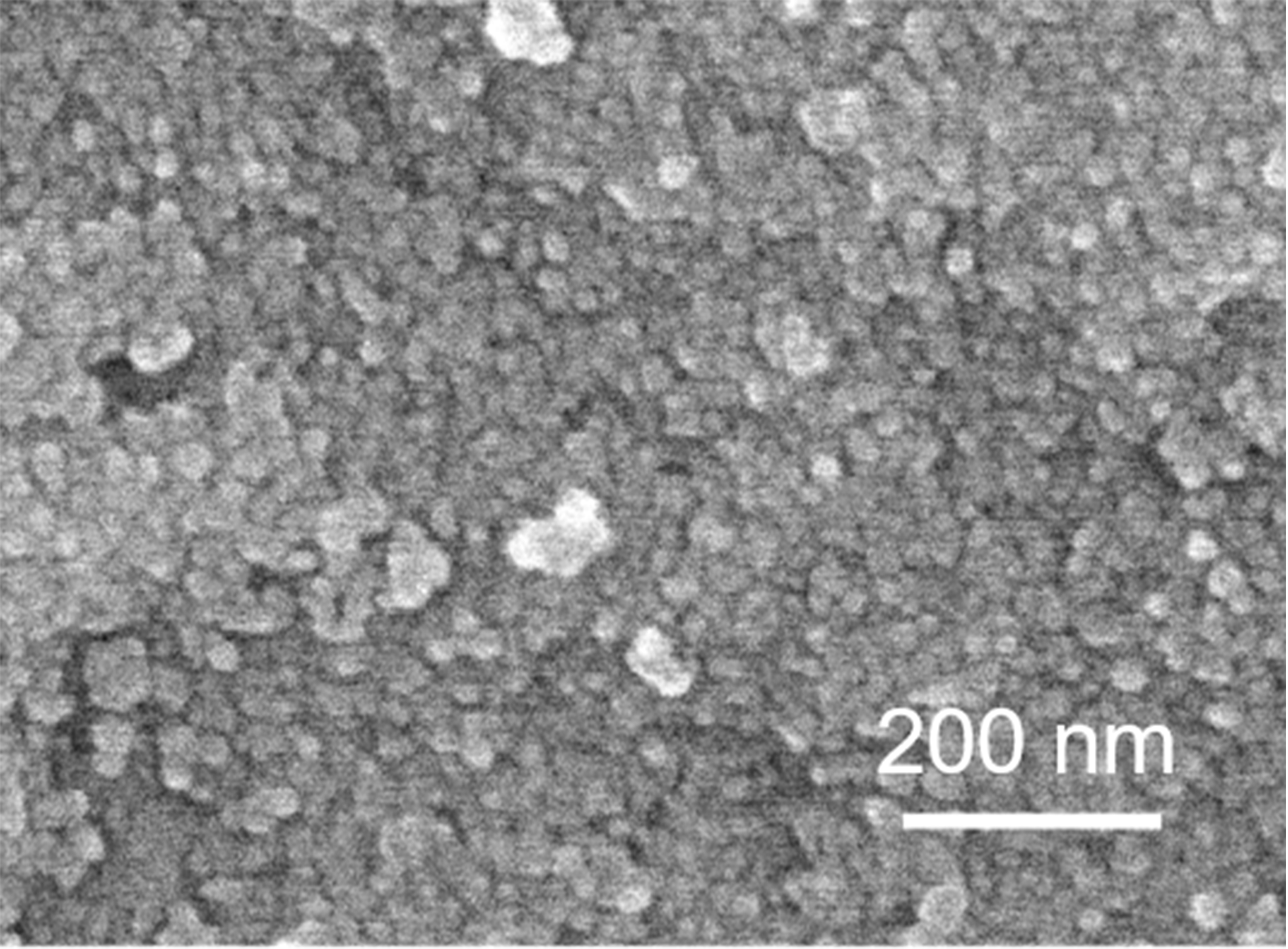


**Figure S7** SEM image of FeHCF. SEM image demonstrates that FeHCF display uniformly sized nanoparticles, which indicates that the massive morphology of NiHCF is constructed by uniform nanoparticles.

**
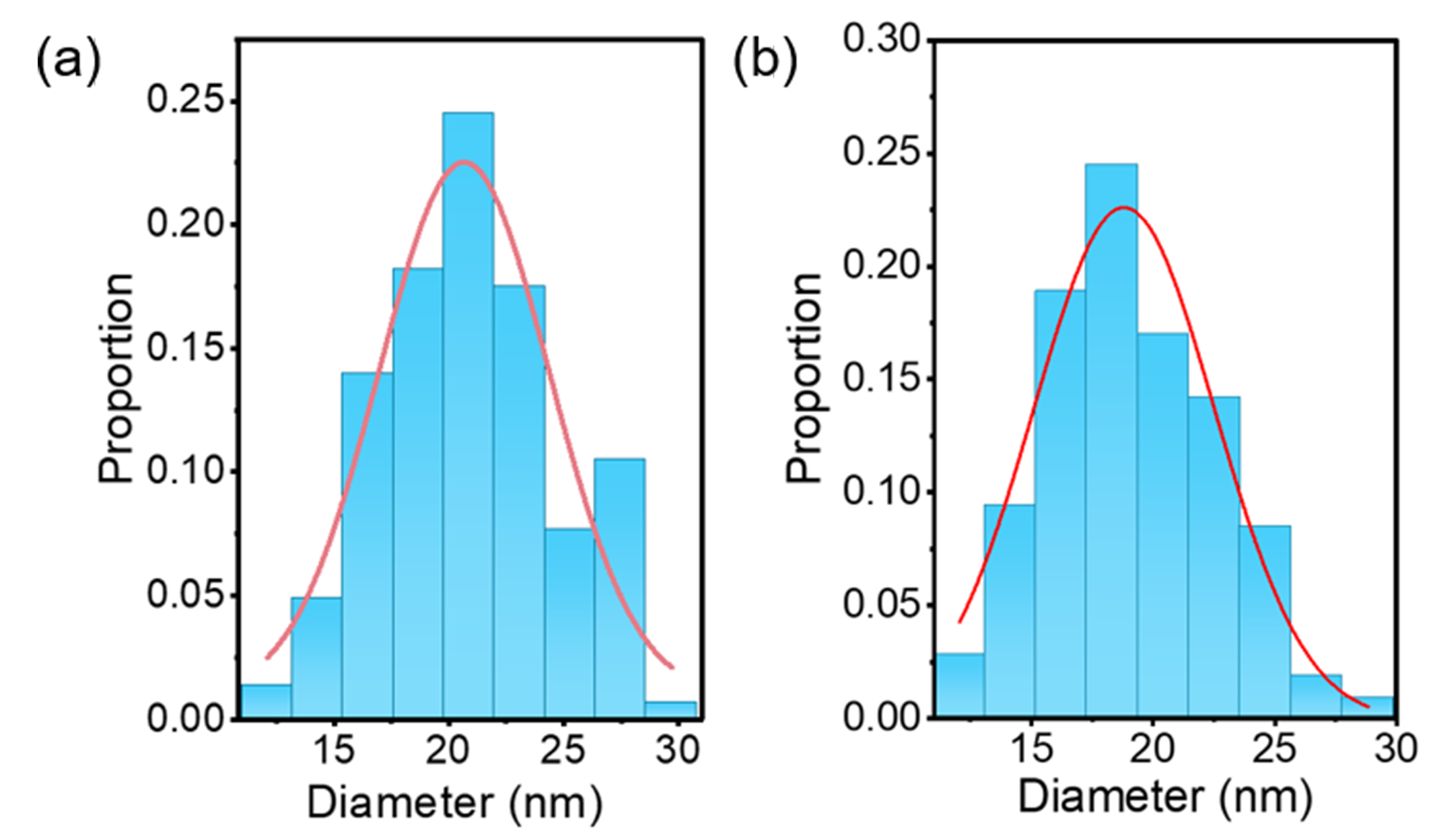
**

**Figure S8** The statistical diagrams of (a) NiHCF and (b) FeHCF according to their related SEM patterns. NiHCF and FeHCF display average particle sizes of 21 nm and 19 nm.


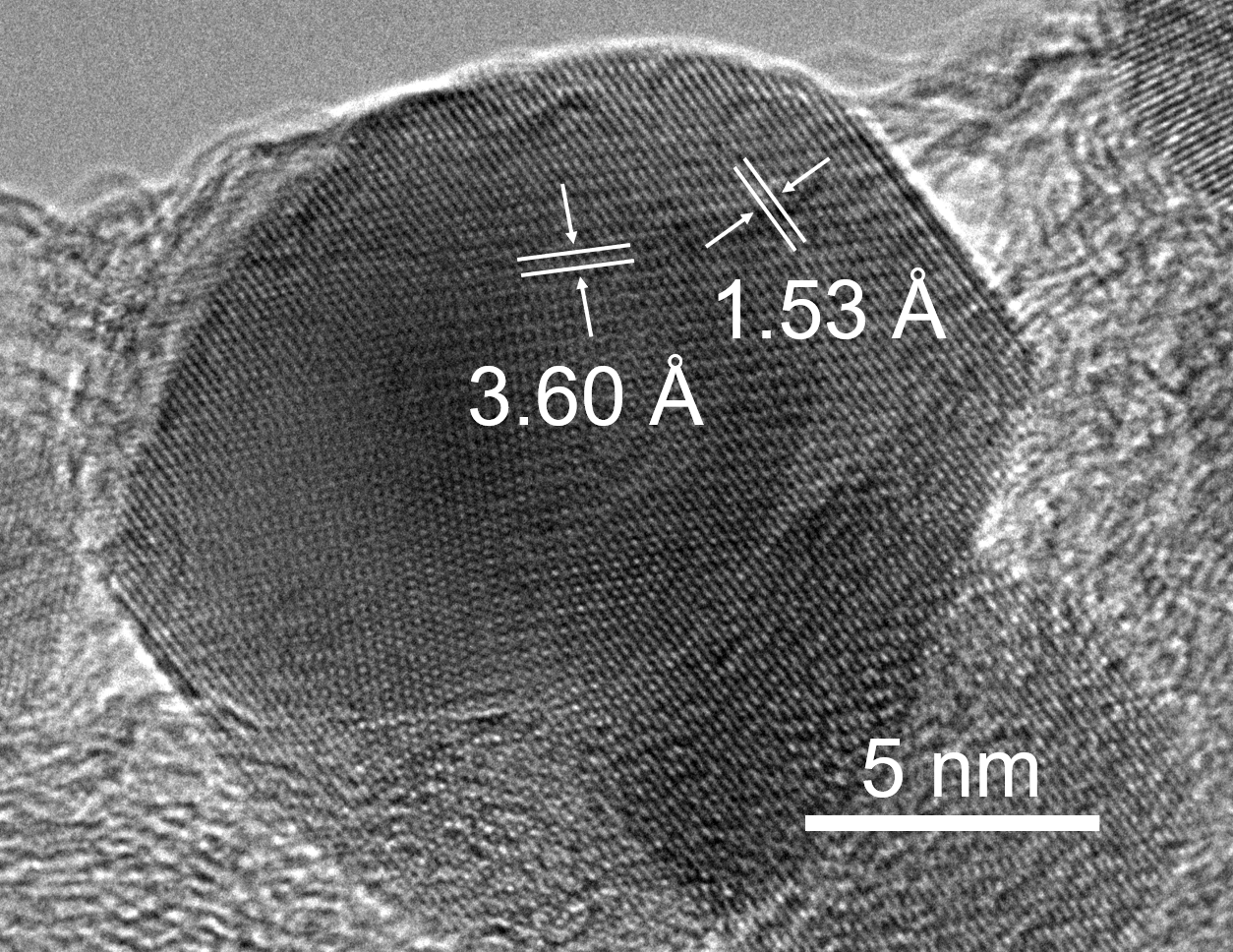


**Figure S9** The HR-TEM patterns of FeHCF. The lattice spacing of 3.60 Å and 1.53 Å, correspond to the (110) and (311) planes of FeHCF, respectively.


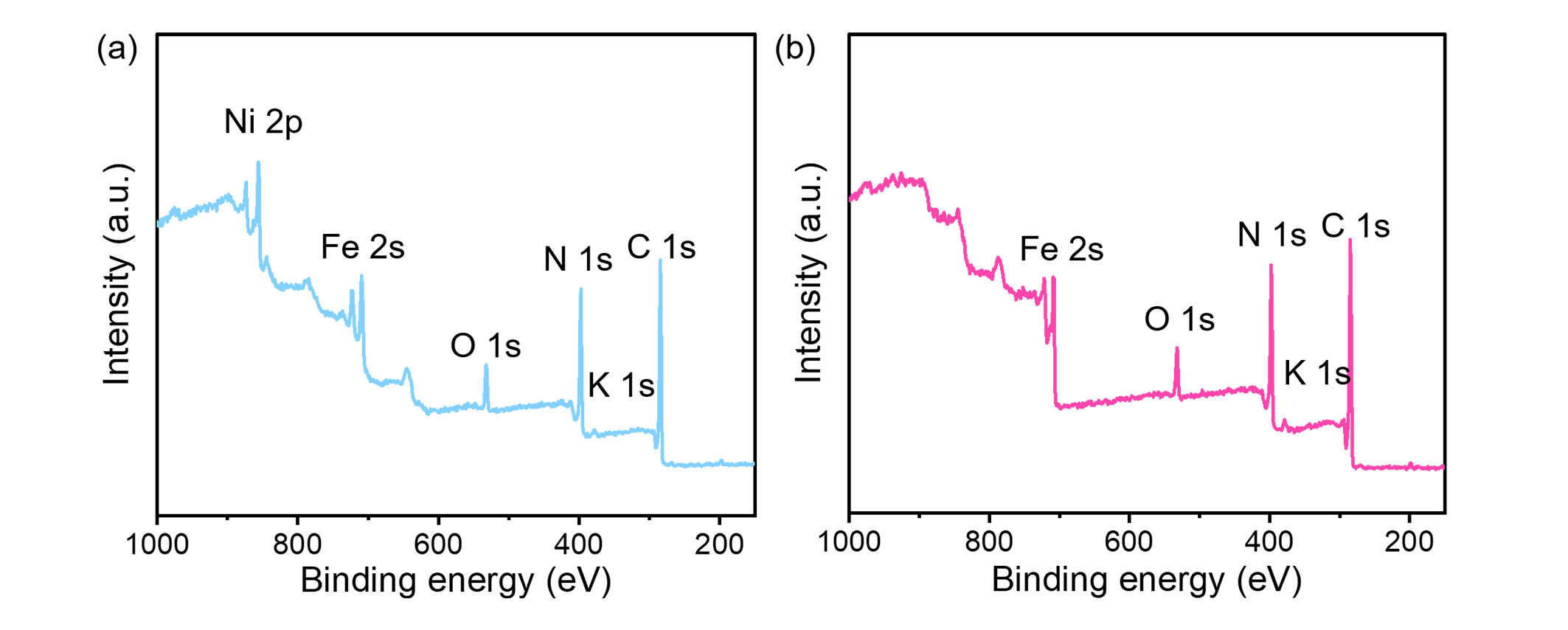


**Figure S10** The full XPS spectra of (a) NiHCF and (b) FeHCF. The full spectra reveal the presence of K, Ni, Fe, C, N, and O elements in NiHCF, and the presence of K, Fe, C, N, and O elements in FeHCF.


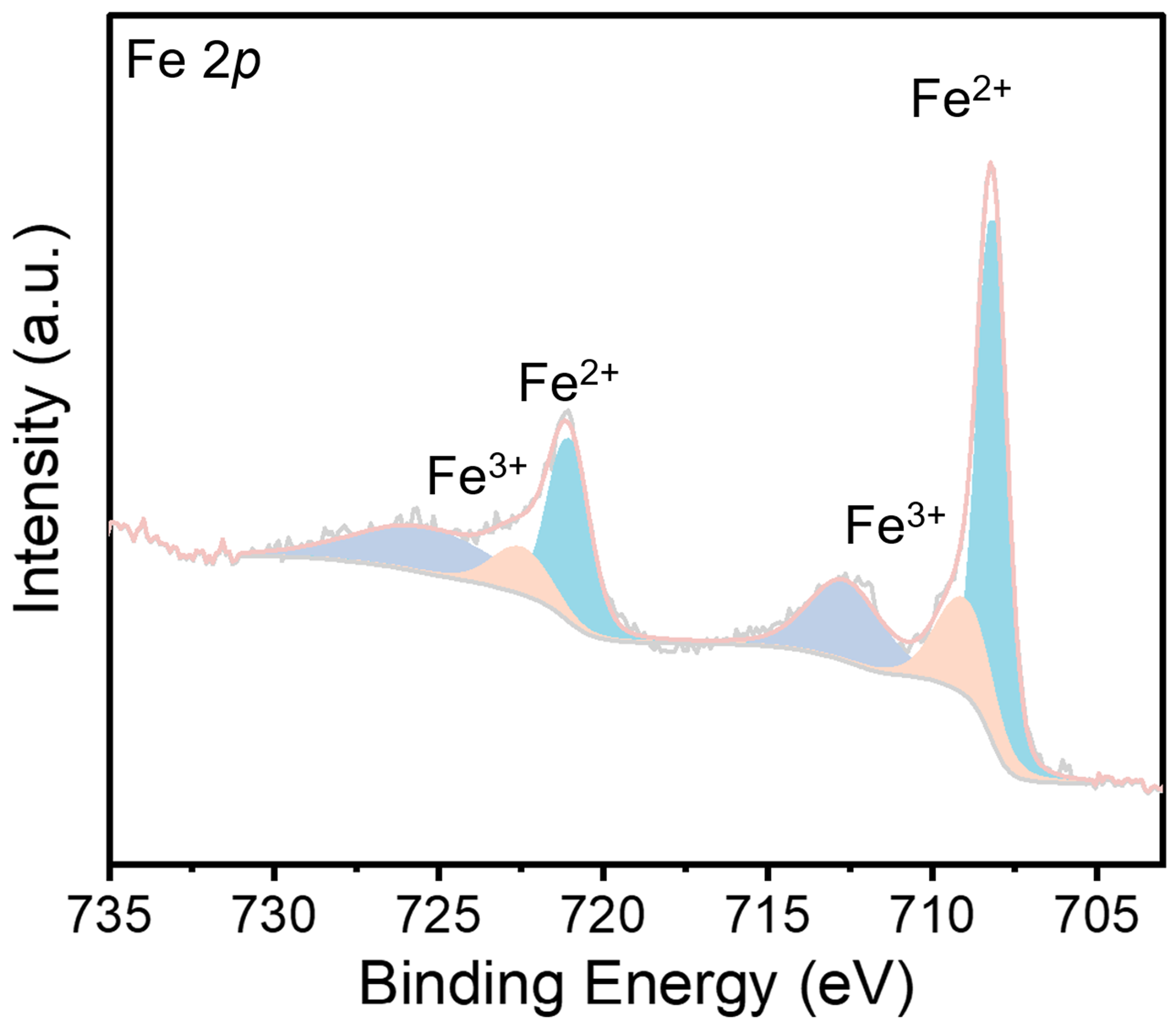


**Figure S11** The Fe 2p XPS spectrum of FeHCF. The characteristic peaks at 708.2 eV and 721.0 eV ascribe to Fe^2+^, while peaks at 709.0 eV and 722.5 eV correspond to Fe^3+^.^[7]^ By calculating the integral area of these characteristic peaks, the ratio of Fe^2+^ to Fe^3+^ ratio is 2.41 and the average valence state of Fe is 2.29.


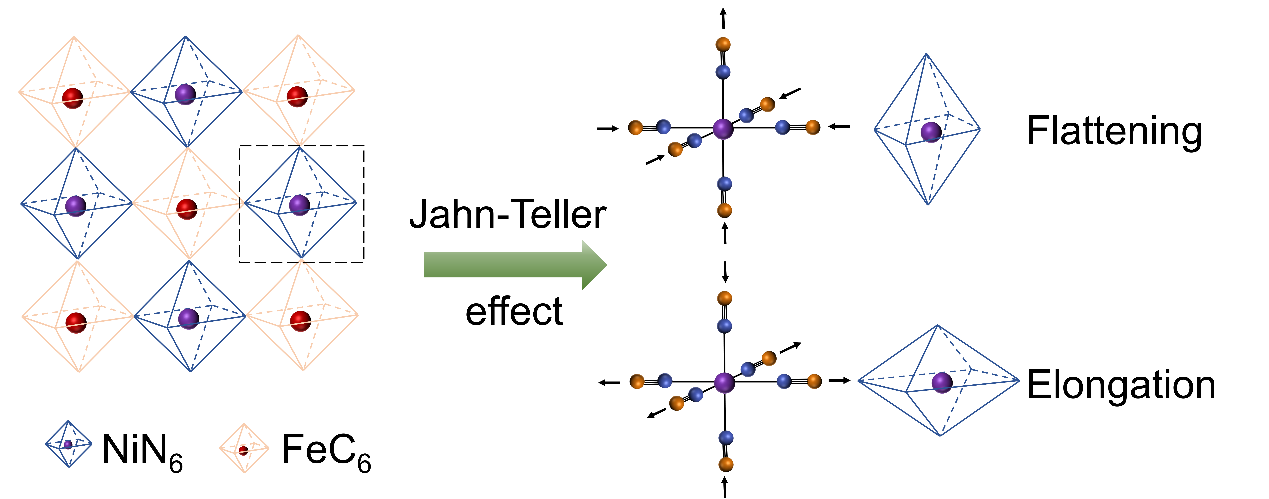


**Figure S12** The schematic diagram of Jahn-Teller effect in NiHCF.


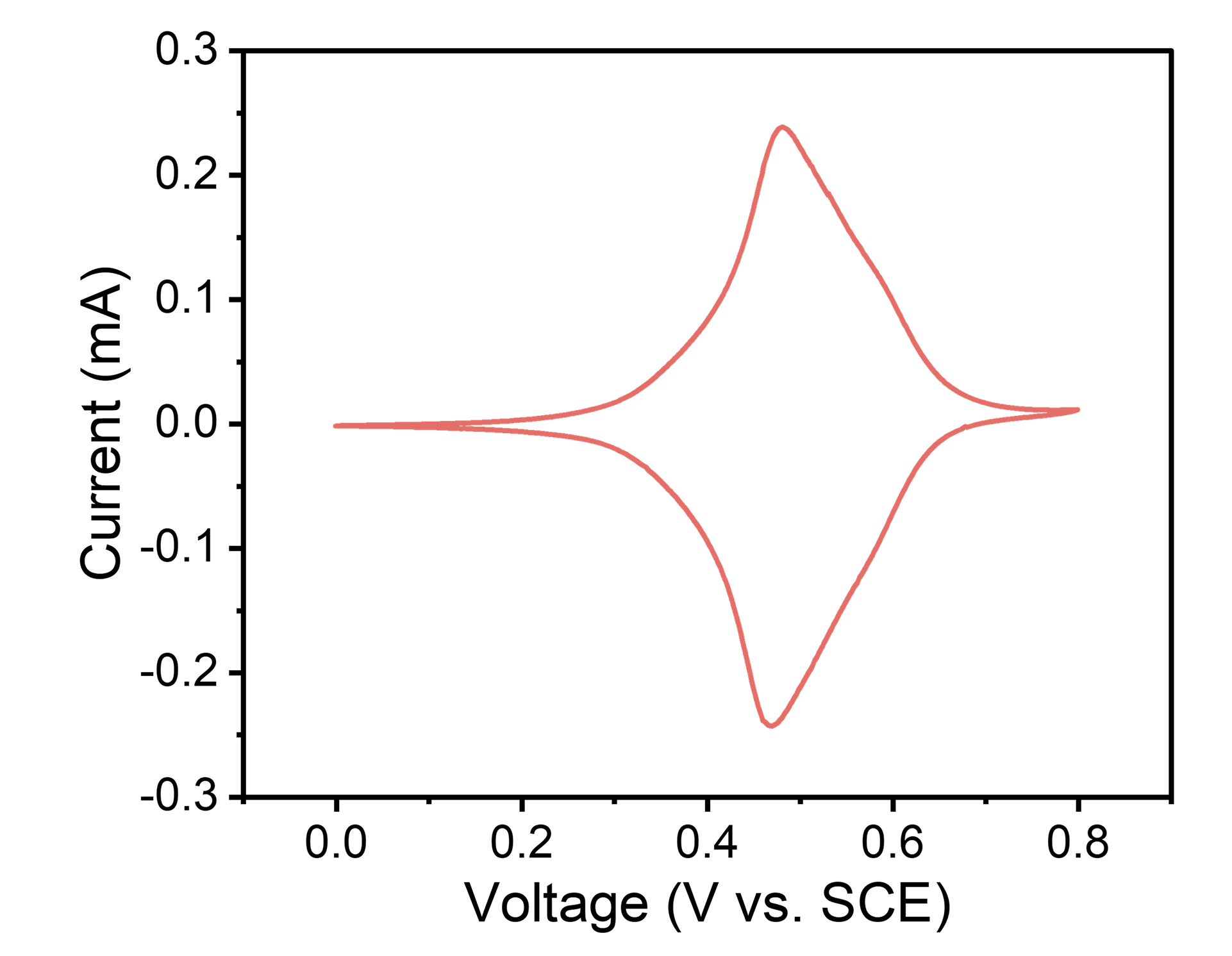


**Figure S13** The pattern of CV curves of NiHCF at the scan rate of 0.2 mV s^-1^ and the potential window of 0-0.8 V (vs. SCE) in aqueous potassium ion batteries. NiHCF displays a couple of redox peaks at 0.47/0.48 V (vs. SCE), which are assigned to Fe^2+^/Fe^3+^ and without any noticeable Ni^2+^/Ni^3+^.


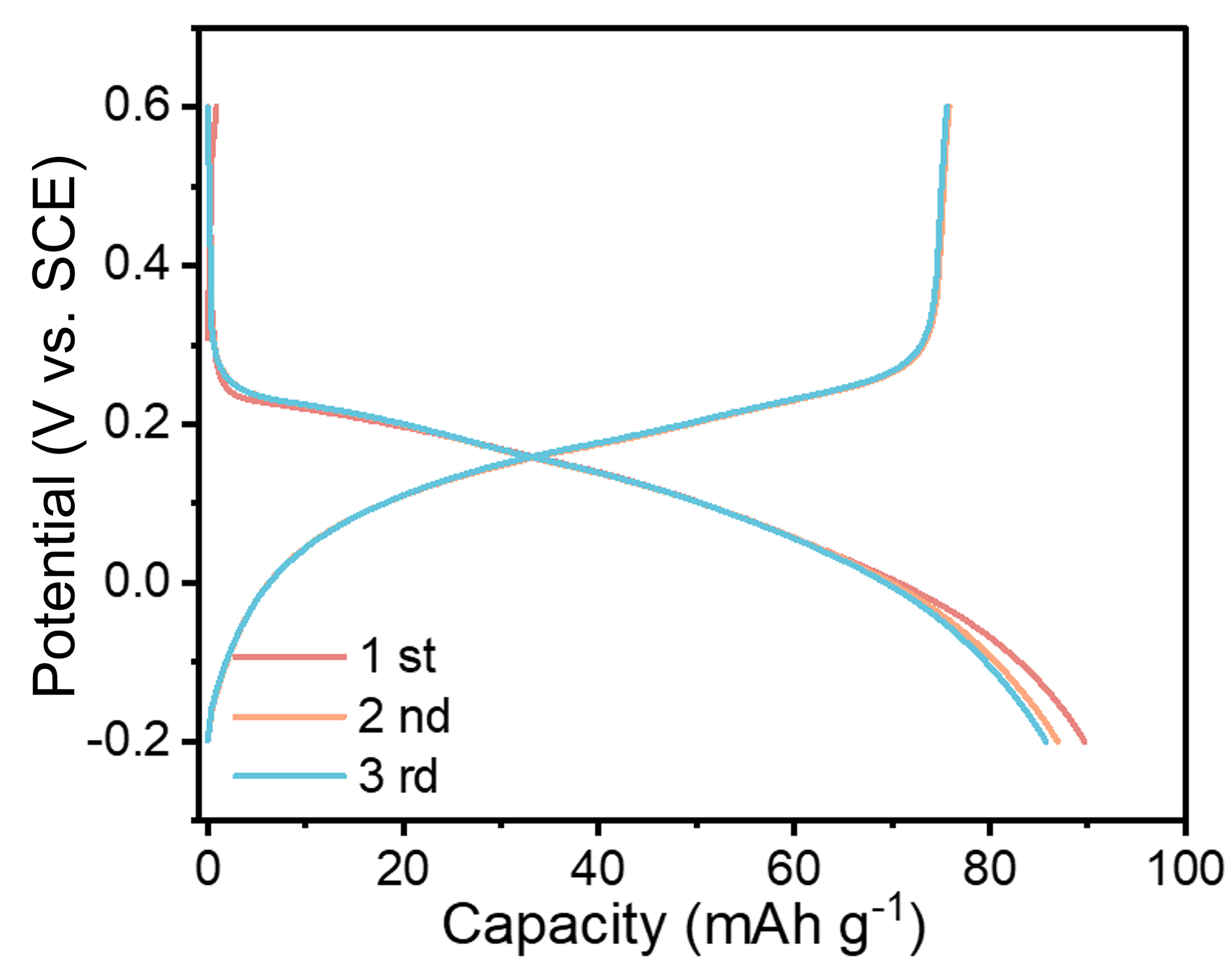


**Figure S14** The first three galvanostatic charging and discharging profiles of FeHCF at the current density of 50 mA g^-1^. FeHCF delivers discharging capacities of 89.6, 86.9, 85.7 mAh g^-1^ with the discharge platform of 0.18 V (vs. SCE).


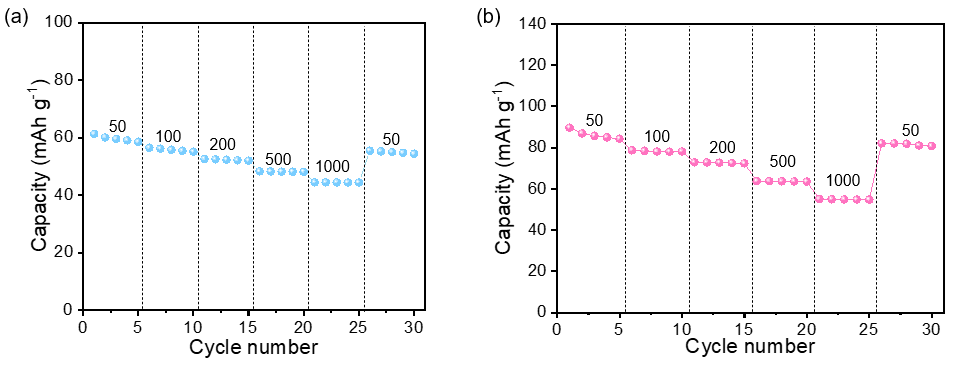


**Figure S15** The rate performance patterns of (a) NiHCF and (b) FeHCF. With the current density increase from 50 to 1000 mA g^-1^, both NiHCF and FeHCF display decreasing capacities and recover their original capacities when current density returns to 50 mA g^-1^.


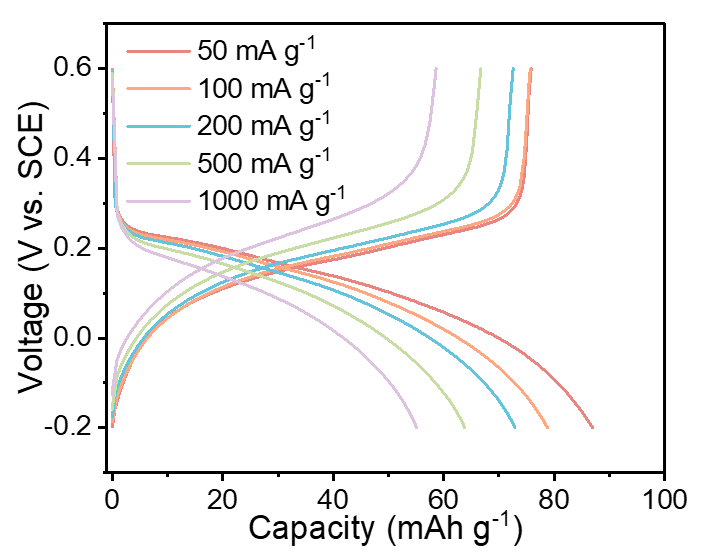


**Figure S16** The galvanostatic charging and discharging curves of FeHCF at different current densities of 50, 100, 200, 500, and 1000 mA g^-1^. FeHCF displays the discharging capacity of 86.9, 78.8, 72.9, 63.8, 55.1 mAh g^-1^ at the current densities of 50, 100, 200, 500, 1000 mA g^-1^, respectively.

**
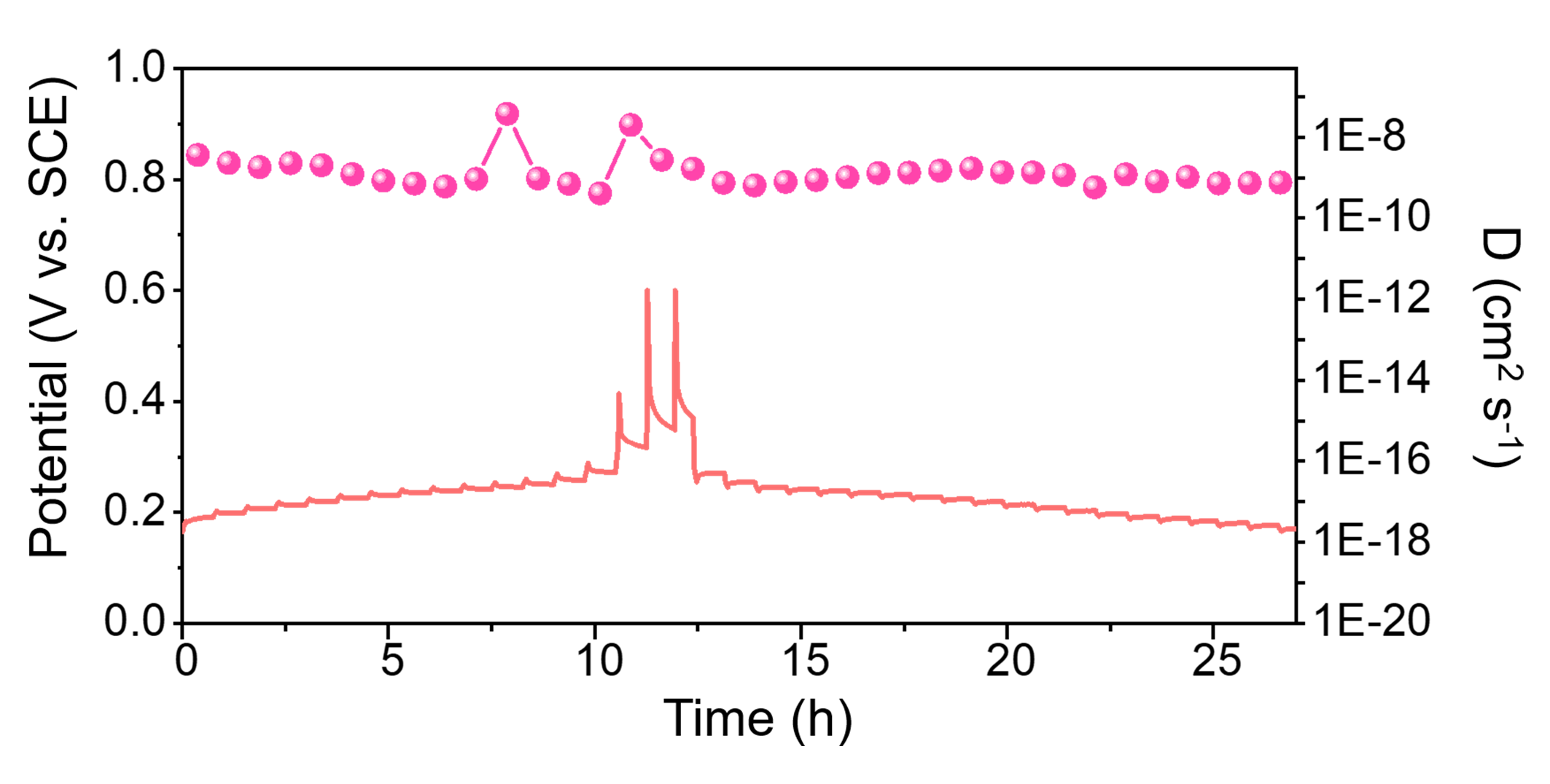
**

**Figure S17** GITT curves and the relevant NH_4_^+^ ion diffusion coefficients (D) for FeHCF. According to calculating through Equation 1, the calculated D values for NiHCF range from 10^-10^ to 10^-8^ cm^2^ s^-1^. However, the D values of FeHCF are under 10^-9^ cm^2^ s^-1^ at the charging and discharging platforms, which is lower than NiHCF.


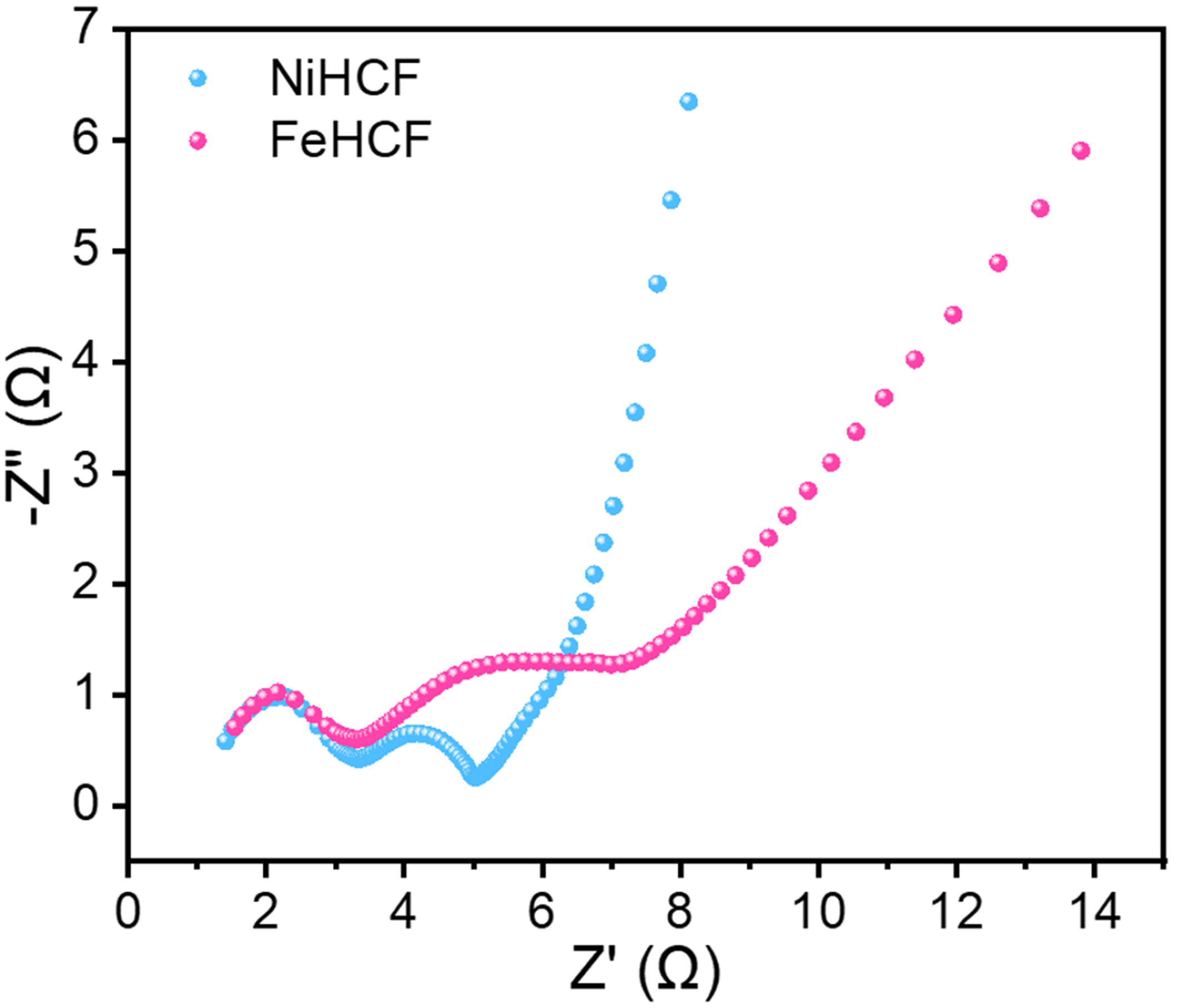


**Figure S18** The EIS curves of NiHCF and FeHCF. NiHCF displays a lower charge transfer resistance (R_ct_) compared to FeHCF, suggesting more efficient electron transfer and faster electrochemical reactions in AIBs.


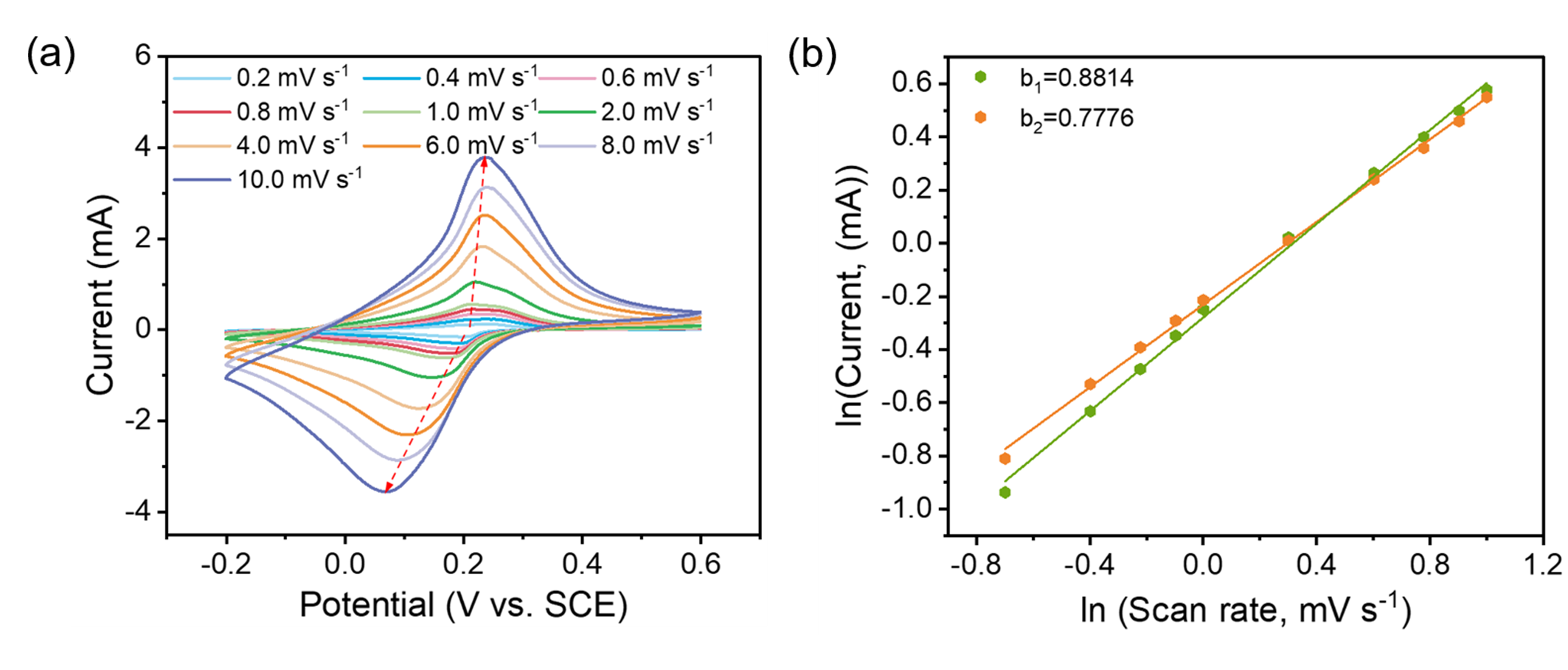


**Figure S19** (a) CV curves of FeHCF at the scan rate of 0.2-10 mV s^-1^. (b) The “b” values based on the redox peaks, where “b_1_” and “b_2_” correspond to oxidation peak and reduction peak in (a). According to Equation 2, the calculated b_1_ and b_2_ are 0.881 and 0.778, indicating that the NH_4_^+^ storage process is controlled by capacitive and diffusive behavior.


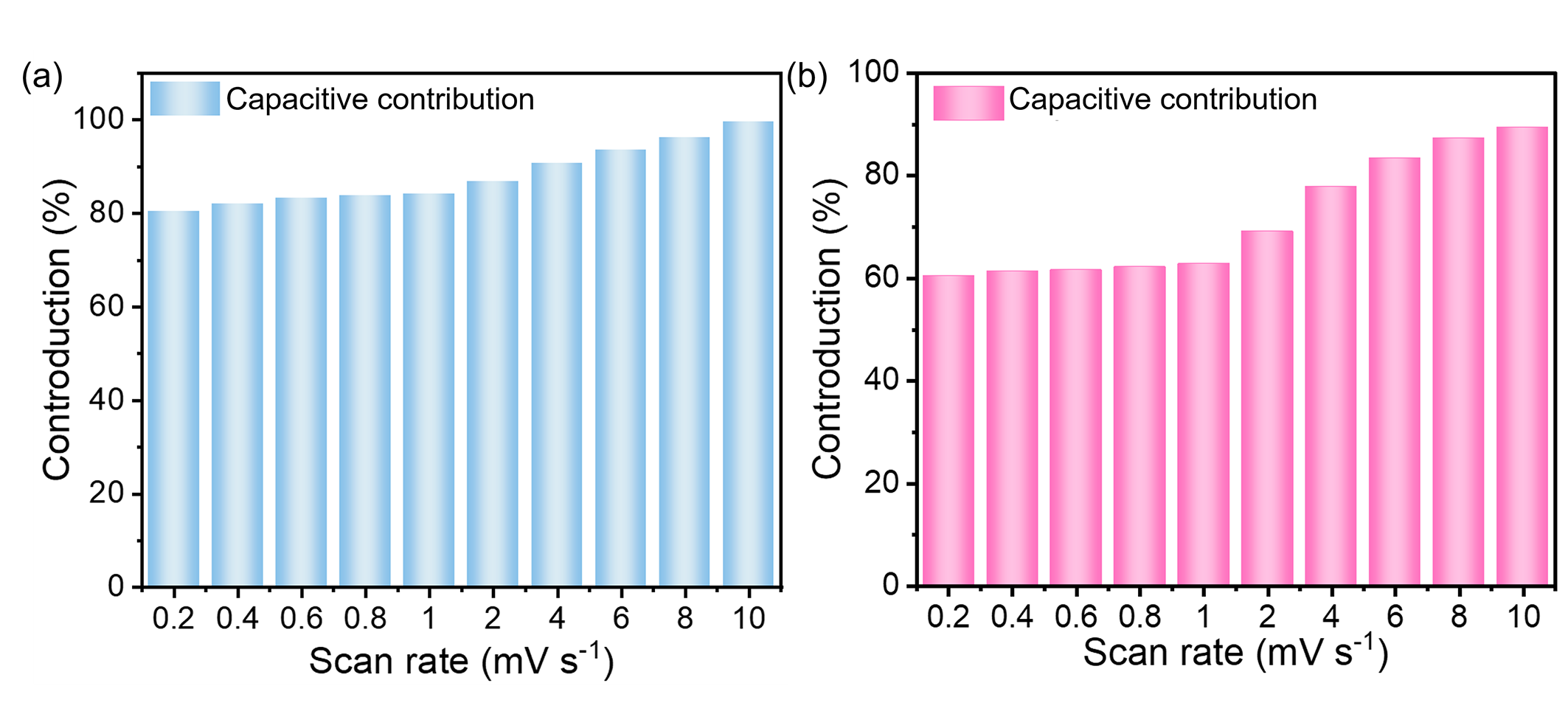


**Figure S20** The capacitive contribution patterns of NiHCF and FeHCF. According to Equation 2, specific capacitive behavior contributions of NiHCF and FeHCF were calculated. The capacitive controlled NH_4_^+^ storage proportion in NiHCF increased from 80.5% to 99.7%, while in FeHCF, it increased from 60.5% to 89.4%.


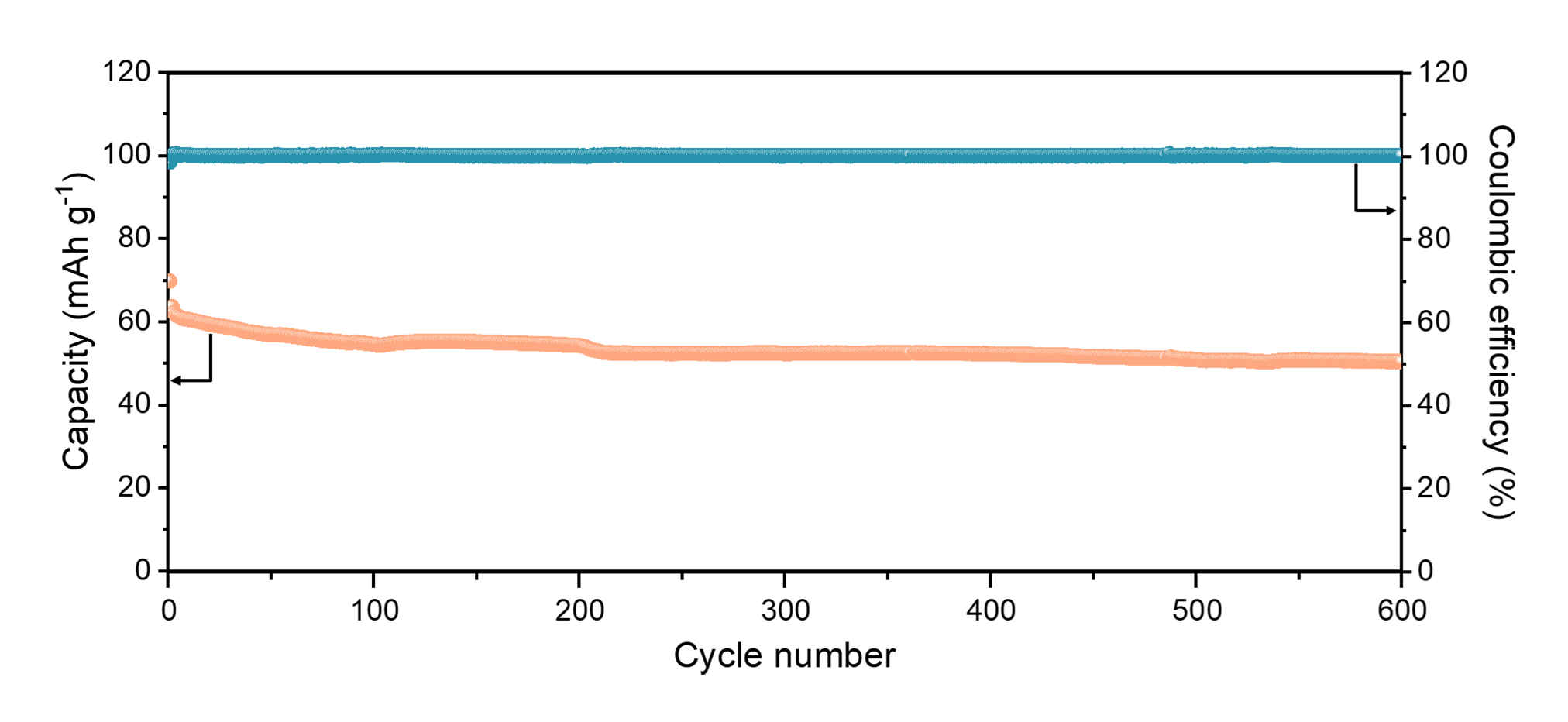


**Figure S21** The cycling performance of FeHCF at the current density of 1000 mA g^-1^. FeHCF retains 72.2% of its initial capacity with nearly 100% coulombic efficiency.


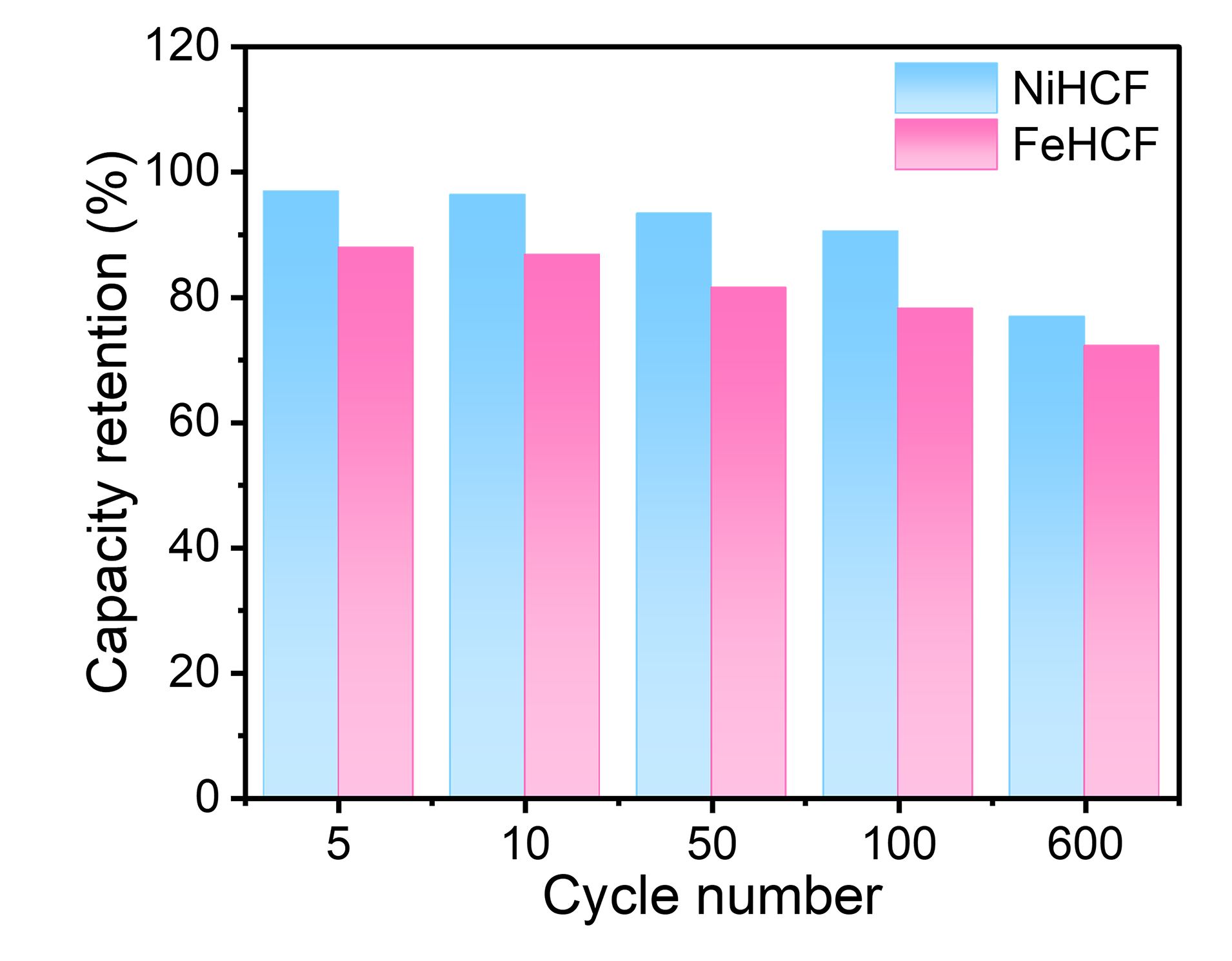


**Figure S22** The capacity retention of NiHCF and FeHCF in different cycles. The capacity retention of NiHCF is higher than that of FeHCF for 5, 10, 50, 100, 600 cycles, indicating better cyclic stability of NiHCF than that of FeHCF.

**
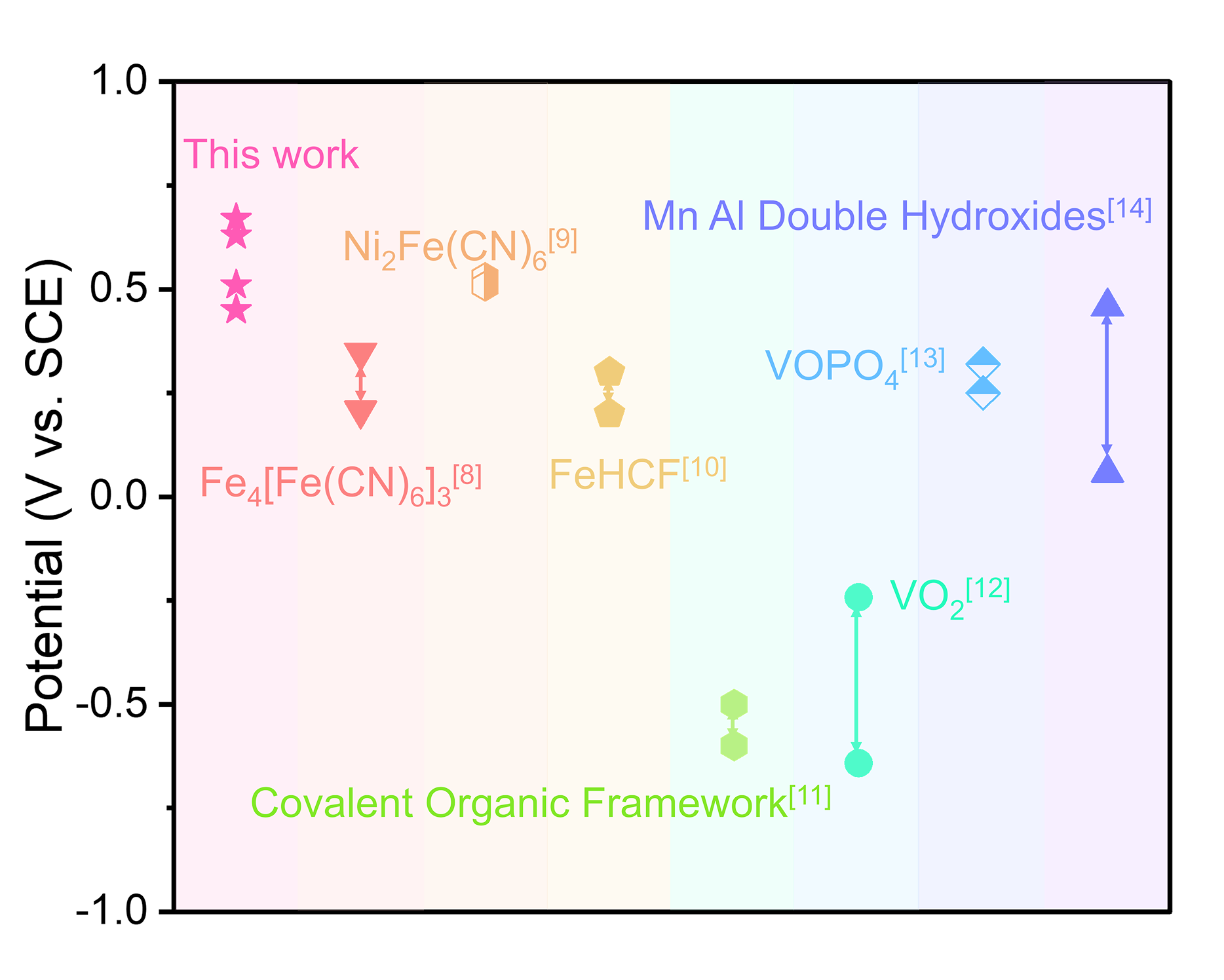
**

**Figure S23** The summary diagram for redox potential of electrode materials for NH_4_^+^ ion storage.^[8-14]^ NiHCF in this work displays higher redox potential than other electrode materials in AIBs.


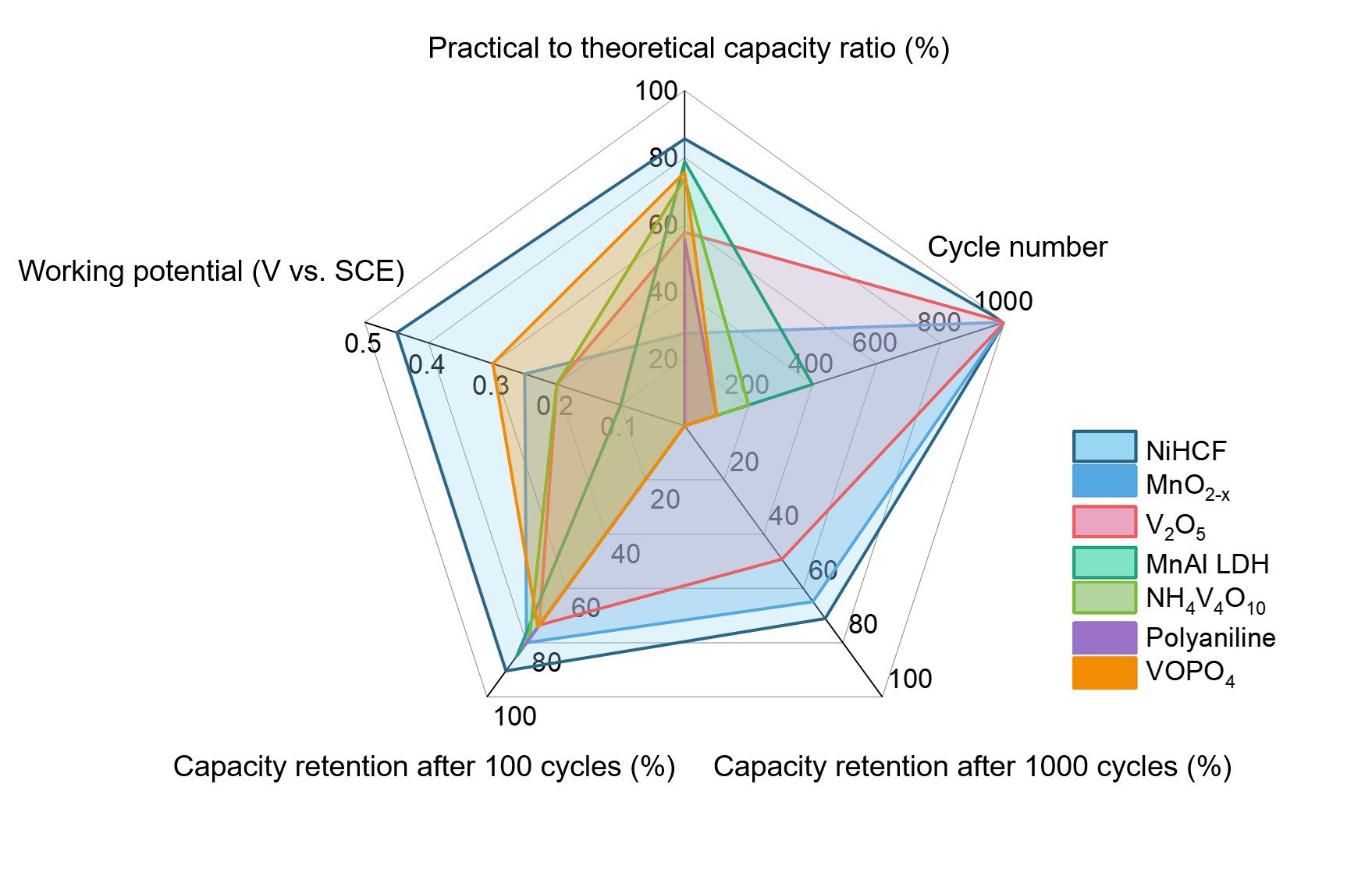


**Figure S24** The comparison diagram among NiHCF in this work and other reported cathode materials. ^[15-20]^


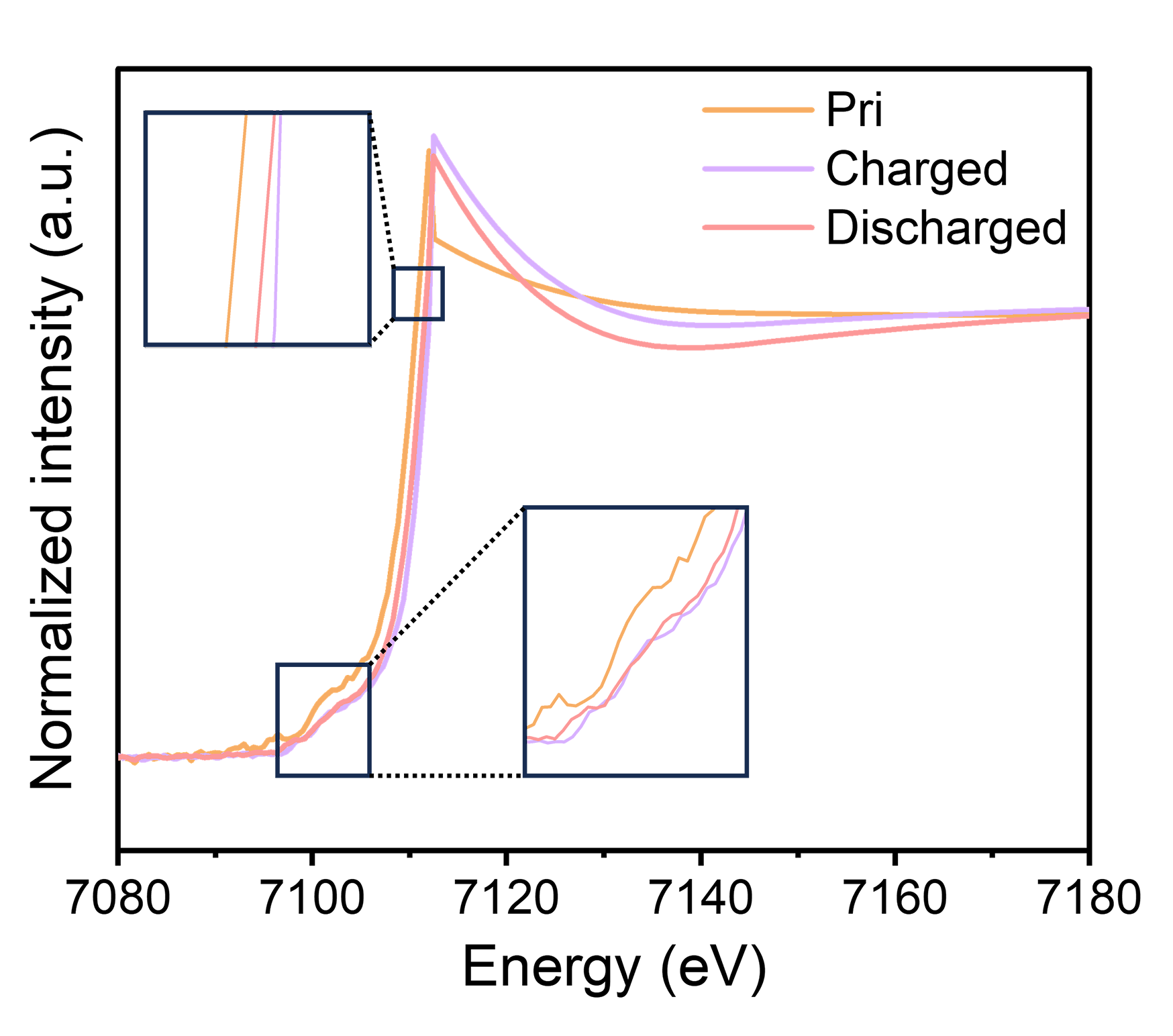


**Figure S25** The Fe K-edge XANES spectra of NiHCF at pristine, charged, and discharged states. Fe K-edge XANES spectra demonstrate that Fe K-edge spectrum at charged state shifts to lower energy, indicating that Fe can provide electrons for NH_4_^+^ during cycling.


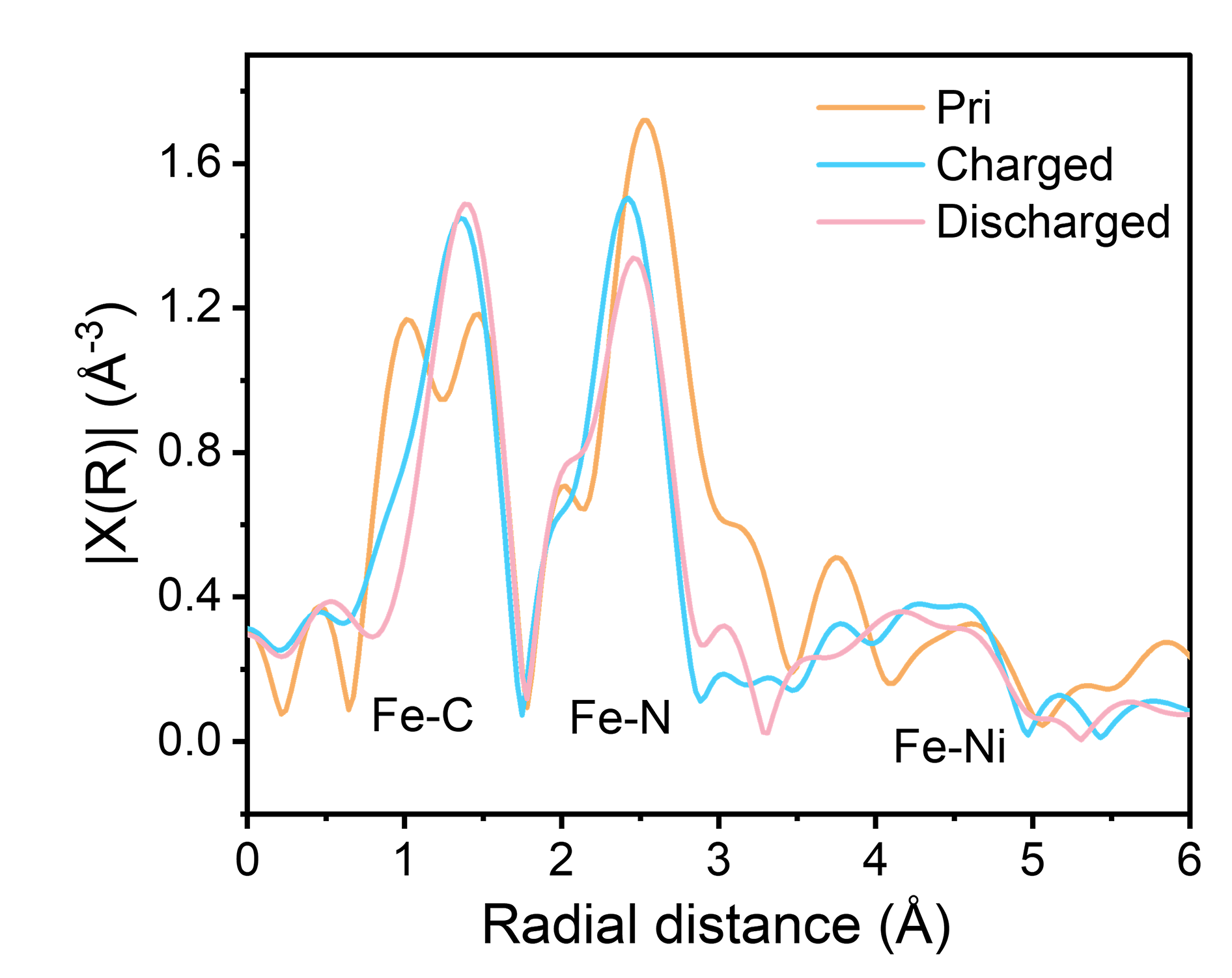


**Figure S26** The Fe K-edge EXAFS spectra R-space curves of NiHCF at pristine, charged, and discharged states, showing atomic distance variations. The main peaks at 1.44 Å, 2.54 Å, and 4.69 Å are attributed to atomic distances of Fe-C, Fe-N, and Fe-Ni, respectively. The peaks of Fe-C and Fe-N at charged state shift to higher atomic distance compared to those at discharged state, which are attributed to the structural distortions of NiHCF.


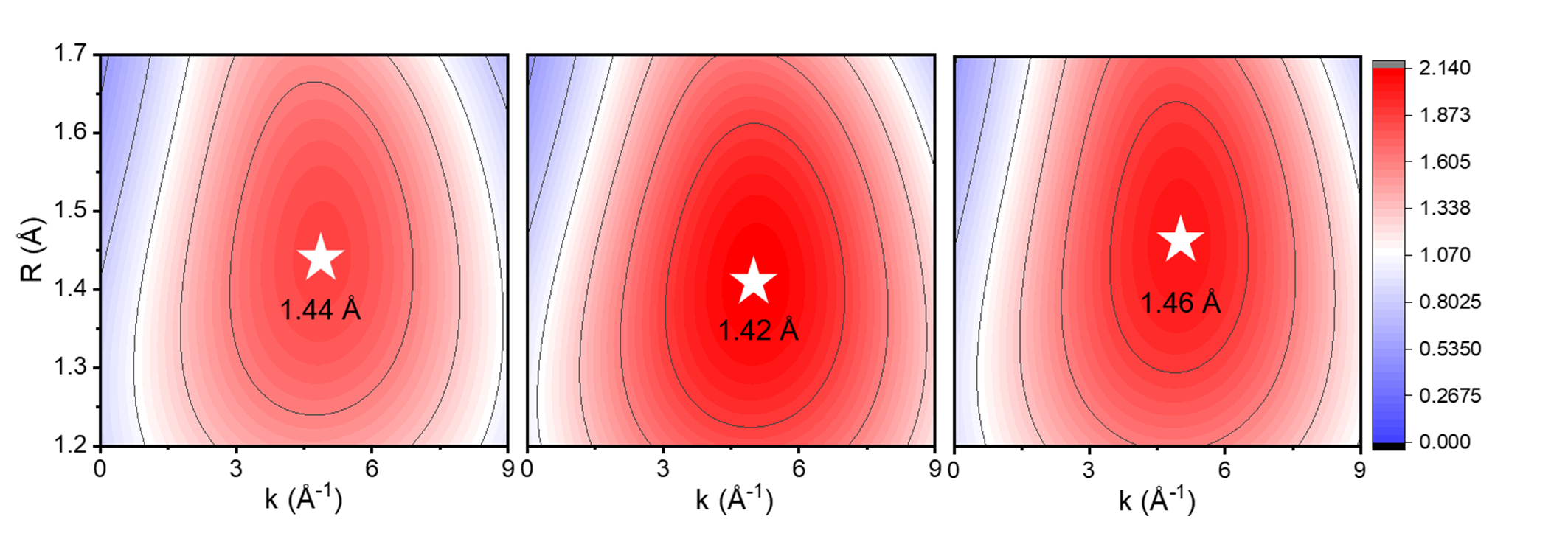


**Figure S27** The Ni K-edge EXAFS wavelet transforms of NiHCF at pristine, charged, and discharged state. The marked sub-lobes correspond to the Ni-N bond of NiHCF. The atomic distances of Ni-N at pristine, charged, discharged state are 1.44, 1.42, and 1.46 Å, respectively.


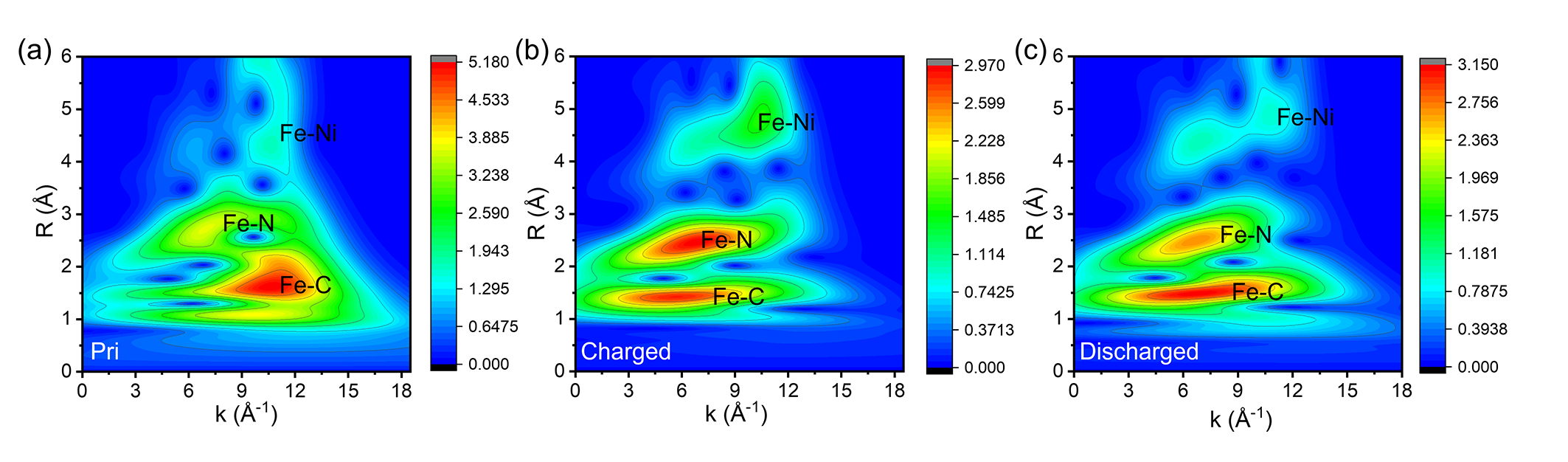


**Figure S28** The Fe K-edge EXAFS wavelet transforms of NiHCF at pristine, charged, and discharged state. The atomic distance of Fe-C at pristine state is larger than those at charged and discharged states. Additionally, the atomic distance of Fe-N at discharged state is higher than those at charged state, which caused by reversible structural variations.


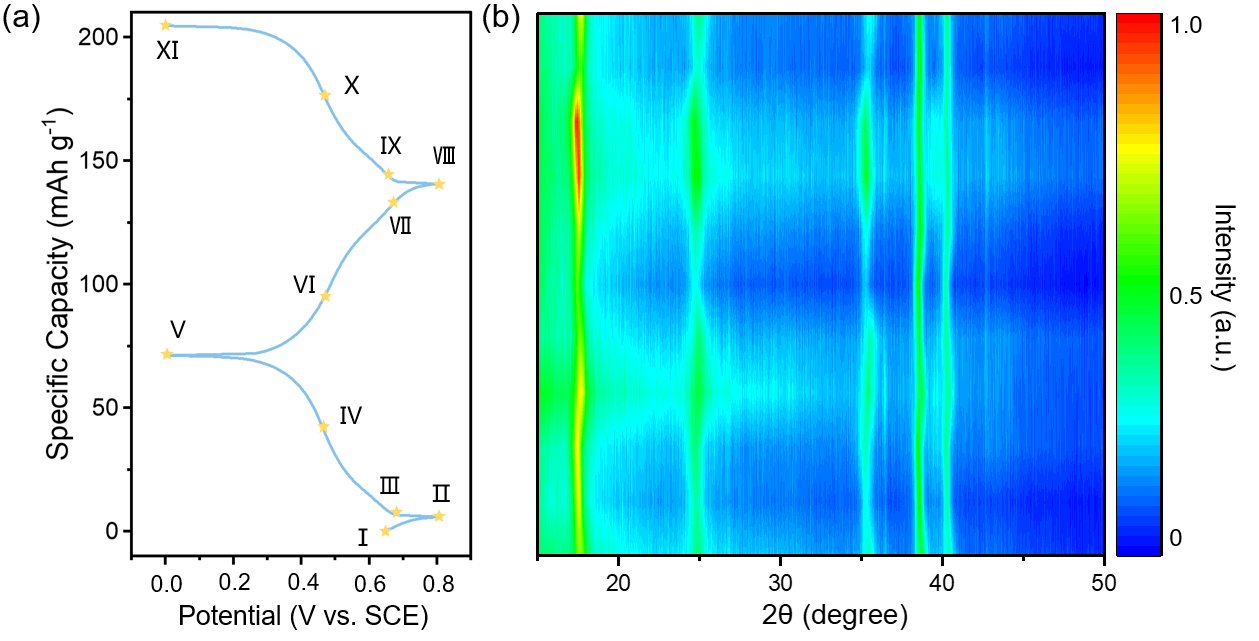


**Figure S29** Ex-situ XRD patterns of NiHCF at different charge-discharge states in AIBs. (a) Galvanostatic charging and discharging profiles. (b) Ex-situ XRD patterns illustrating crystallinity variations corresponding to the intercalation and de-intercalation of NH_4_^+^.

2. **Supplemental Tables**

**Table S1** The crystalline information of NiHCF.

| **Crystal System** | Cubic crystal space |
| --- | --- |
| **Space Group** | Fm-3m |
| **Lattice Constants** | a=b=c=10.19 Å; α=β=γ=90º |
| **Unit Cell Volume** | 1060 Å^3^ |
| **Ni-N Bond Length** | 2.07 Å |
| **Ni-N≡C Bond Angle** | 180º |

**Table S2** The crystalline information of FeHCF.

| **Crystal System** | Cubic crystal space |
| --- | --- |
| **Space Group** | Fm-3m |
| **Lattice Constants** | a=b=c=10.18 Å; α=β=γ=90º |
| **Unit Cell Volume** | 1053 Å^3^ |
| **Fe-N Bond Length** | 2.10 Å |
| **Fe-N≡C Bond Angle** | 180º |

**Table S3** The contents of K, Fe, Ni elements for NiHCF and FeHCF tested by ICP.

|  | NiHCF (wt.%) | FeHCF (wt.%) |
| --- | --- | --- |
| K | 1.38 | 2.80 |
| Fe | 9.50 | 33.81 |
| Ni | 14.15 | 0 |

**Table S4** The element contents of NiHCF tested by EDS mapping.

| Element | Weight (%) | Atomic (%) |
| --- | --- | --- |
| C | 36.12 | 51.79 |
| N | 31.07 | 38.20 |
| K | 1.45 | 0.64 |
| Fe | 11.54 | 3.56 |
| Ni | 19.82 | 5.82 |

**Table S5** The element contents of FeHCF tested by EDS mapping.

| Element | Weight (%) | Atomic (%) |
| --- | --- | --- |
| C | 39.62 | 54.65 |
| N | 30.60 | 36.20 |
| K | 2.44 | 1.03 |
| Fe | 27.34 | 8.11 |

**Table S6** Comparison of the discharge capacities and theoretical capacities for Prussian blue and its analogues in AIBs.

| **Materials** | **Capacity** | **Theoretical capacity** | **Ratio** | **Reference** |
| --- | --- | --- | --- | --- |
| **NiHCF** | **61.3 mA h g^-1^ at 50 mA g^-1^** | **76.1 mAh g^-1^** | **85.6%** | **This work** |
| Fe_4_[Fe(CN)_6_]_3_ | 61.7 mAh g^-1^ at 1 C | 187.0 mAh g^-1^ | 33.0% | ^[8]^ |
| FeFe(CN)_6_ | 80.0 mAh g^-1^ at 30 mA g^-1^ | 200.0 mAh g^-1^ | 40.0% | ^[21]^ |
| NaFeFe(CN)_6_ | 62.0 mA h g^-1^ at 250 mA g^-1^ | 184.2 mAh g^-1^ | 33.7% | ^[22]^ |
| N-CuHCF | 60.0 mAh g^-1^ at 1 C | 200.2 mAh g^-1^ | 30.0% | ^[23]^ |
| CuHCF | 74.9 mAh g^-1^ at 100 mA g^-1^ | 181.4 mAh g^-1^ | 41.3% | ^[11]^ |
| Cu_0.4_Ni_1.6_Fe(CN)_6_ | 52.4 mAh g^-1^ at 0.3 C | 161.7 mAh g^-1^ | 32.4% | ^[24]^ |
| Ni_2_Fe(CN)_6_ | 57.8 mAh g^-1^ at 1 C | 81.4 mAh g^-1^ | 71.0% | ^[25]^ |
| Ni_2_Fe(CN)_6_ | 60.0 mAh g^-1^ at 1 C | 81.4 mAh g^-1^ | 73.7% | ^[10]^ |
| Ni-APW | 62.6 mAh g^-1^ 150 mA g^-1^ | 86.8 mAh g^-1^ | 72.1% | ^[26]^ |
| Ni_2_Fe(CN)_6_ | 60.3 mAh g^-1^ at 1 C | 81.4 mAh g^-1^ | 74.1% | ^[9]^ |

**Table S7** Comparison of the discharge capacities for cathode materials in AIBs.

| **Materials** | **Capacity** | **Reference** |
| --- | --- | --- |
| **NiHCF** | **61.3 mA h g^-1^ at 50 mA g^-1^** | **This work** |
| MnO_2-x_ | 80.5 mA h g^-1^ at 1000 mA g^-1^ | ^[15]^ |
| V_2_O_5_ | 170.0 mA h g^-1^ at 500 mA g^-1^ | ^[16]^ |
| MnAl layered double hydroxide | 183.7 mA h g^-1^ at 100 mA g^-1^ | ^[19]^ |
| NH_4_V_4_O_10_ | 155.5 mA h g^-1^ at 50 mA g^-1^ | ^[18]^ |
| Polyaniline | 160.0 mA h g^-1^ at 1000 mA g^-1^ | ^[17]^ |
| VOPO_4_ | 125.1 mA h g^-1^ at 100 mA g^-1^ | ^[20]^ |

**Table S7** presents a comparative analysis of discharge capacities for various cathode materials in AIBs. This indicates that NiHCF does not lead in terms of absolute practical capacity among the evaluated cathode materials. Despite this limitation, NiHCF displays a superior practical-to-theoretical capacity ratio compared to other materials (**Figure** **S24**), which directly reflects efficient capacity utilization and minimized irreversible side reactions during NH_4_^+^ storage. These attributes enhance the viability of NiHCF for applications where efficient capacity utilization and long-term cycling stability are critical.

**Table S8** The DFT calculation of absorption for NiHCF and FeHCF.

|  | NiHCF (eV) | FeHCF (eV) |
| --- | --- | --- |
| E_slab_ | -451.30 | -470.11 |
| E_ammonium_ | -20.87 | -20.87 |
| E_complex_ | -477.44 | -495.78 |
| E_absorption_ | -5.26 | -4.80 |

**Table S9** The lattice spacing of NiHCF at charged and discharged states.

| **Lattice plane** | **(100)** | **(110)** | **(200)** |
| --- | --- | --- | --- |
| **Charged state** | 5.06 Å | 3.59 Å | 2.54 Å |
| **Discharged state** | 5.00 Å | 3.57 Å | 2.52 Å |

**Table S10** The comparison of electrochemical performance for ammonium ion full cell.

| **Full cell** | **Capacity** | **Energy density** | **Cpacity retention** | **Reference** | |
| --- | --- | --- | --- | --- | --- |
| **NiHCF//AC** | **62.2 mA h g^-1^ at 50 mA g^-1^** | **56.0 Wh kg^-1^** | **100% after 1000 cycles at 1000 mA g^-1^** | | **This work** |
| NH_4_Fe_4_[Fe(CN)_6_]_3_//PTCDI | 54.3 mAh g^-1^ at 1 C | 12.3 Wh kg^-1^ | 89.8% after 300 cycles at 1 C | | ^[8]^ |
| Ni-APW//PTCDI | 41.0 mAh g^-1^ at 60 mA g^-1^ | 32.8 Wh kg^-1^ | 67.0% after1000 cycles at 120 mA g^-1^ | | ^[26]^ |
| FeHCF//CuHCF | 55 mAh g^-1^ at 200 mA g^-1^ | 34.8 Wh kg^-1^ | 28.9% after 500 cycles at 200 mA g^-1^ | | ^[10]^ |
| PNFF-60/PI@MXene | 43.6 mA h g^-1^ at 1000 mA g^-1^ | 34.9 Wh kg^-1^ | 83.0% after 200 cycles at 1000 mA g^-1^ | | ^[27]^ |

1. **Supplementary Reference**

[1] S. Li, Y. Liu, X. Zhao, K. Cui, Q. Shen, P. Li, X. Qu, L. Jiao, *Angewandte Chemie International Edition* **2021**, 60, 20286.

[2] S. Li, Y. Liu, X. Zhao, Q. Shen, W. Zhao, Q. Tan, N. Zhang, P. Li, L. Jiao, X. Qu, *Advanced Materials* **2021**, 33, 2007480.

[3] Y. Zhao, P. Zhang, J. Liang, X. Xia, L. Ren, L. Song, W. Liu, X. Sun, *Energy Storage Materials* **2022**, 47, 424.

[4] G. Moretti, C. Gervais, *Journal of Raman Spectroscopy* **2018**, 49, 1198.

[5] J. Vázquez‐Samperio, N. Sánchez‐Padilla, P. Acevedo‐Peña, A. Cano, N. Nava, D. Morales‐Acosta, M. Oliver‐Tolentino, *ChemistrySelect* **2018**, 3, 11441.

[6] Y. Xu, M. Chang, C. Fang, Y. Liu, Y. Qiu, M. Ou, J. Peng, P. Wei, Z. Deng, S. Sun, X. Sun, Q. Li, J. Han, Y. Huang, *ACS Applied Materials & Interfaces* **2019**, 11, 29985.

[7] R. Hua, C. Xu, H. Yang, D. Qu, R. Zhang, D. Liu, H. Tang, J. Li, D. Qu, *ACS Applied Materials & Interfaces* **2024**, 16, 20520.

[8] M. Xia, X. Zhang, H. Yu, Z. Yang, S. Chen, L. Zhang, M. Shui, Y. Xie, J. Shu, *Chemical Engineering Journal.* **2021**, 421, 127759.

[9] H. Yu, J. Xu, C. Deng, M. Xia, X. Zhang, J. Shu, Z. Wang, *ACS Applied Energy Materials* **2021**, 4, 9594.

[10] Z. Zhao, W. Zhang, M. Liu, D. Wang, X. Wang, L. Zheng, X. Zou, Z. Wang, D. Li, K. Huang, W. Zheng, *Energy & Environmental Materials* **2022**, 0, 1.

[11] X. Zhang, M. Xia, H. Yu, J. Zhang, Z. Yang, L. Zhang, J. Shu, *Nano Micro Letters* **2021**, 13, 139.

[12] J. Gong, P. Bai, Y. Zhang, Z. Zhou, Q. Wang, H. Lv, T. Hu, X. Wu, C. Meng, *Small* **2024** 21, e2408467.

[13] X. Zhang, H. Wei, B. Ren, J. Jiang, G. Qu, J. Yang, G. Chen, H. Li, C. Zhi, Z. Liu, *Advanced Materials* **2023**, 35, e2304209.

[14] Q. Liu, F. Ye, K. Guan, Y. Yang, H. Dong, Y. Wu, Z. Tang, L. Hu, *Advanced Energy Materials* **2023**, 13, 2202908.

[15] T. Lu, C. Zeng, H. Zhang, X. Shi, Y. Yu, X. Lu, *Small* **2023**, 19, e2206727.

[16] L. Xing, H. Chen, X. Wen, W. Zhou, K. Xiang, *Journal of Alloys and Compounds* **2022**, 925, 166652.

[17] S. Kuchena, Y. Wang, *ACS Applied Energy Materials* **2020**, 3, 11690.

[18] X. Bai, J. Yang, F. Zhang, Z. Jiang, F. Sun, C. Pan, H. Di, S. Ru, D. Liao, H. Zhang, *Dalton Transactions* **2023**, 52, 4923.

[19] Q. Liu, F. Ye, K. Guan, Y. Yang, H. Dong, Y. Wu, Z. Tang, L. Hu, *Advanced Energy Materials* **2023**, 13, 2202908.

[20] F. Ye, R. Pang, C. Lu, Q. Liu, Y. Wu, R. Ma, L. Hu, *Angewandte Chemie International Edition* **2023**,62, e202303480.

[21] S. Li, M. Xia, C. Xiao, X. Zhang, H. Yu, L. Zhang, J. Shu, *Dalton Transactions* **2021**, 50, 6520.

[22] C. Li, W. Yan, S. Liang, P. Wang, J. Wang, L. Fu, Y. Zhu, Y. Chen, Y. Wu, W. Huang, *Nanoscale Horizons* **2019**, 4, 991.

[23] X. Zhang, M. Xia, T. Liu, N. Peng, H. Yu, R. Zheng, L. Zhang, M. Shui, J. Shu, *Chemical Engineering Journal* **2020**, 421, 127767.

[24] L. Fan, G. Shu, Y. Liu, H. Yu, L. Yan, L. Zhang, J. Shu, *Journal of Materials Science & Technology* **2024**, 169, 19.

[25] H. Yu, L. Fan, H. Yan, C. Deng, L. Yan, J. Shu, Z. Wang, *Inorganic Chemistry Frontiers* **2022**, 9, 2001.

[26] X. Wu, Y. Qi, J. Hong, Z. Li, A. Hernandez, X. Ji, *Angewandte Chemie International Edition* **2017**, 56, 13026.

[27] W. Hou, C. Yan, P. Shao, K. Dai, J. Yang, *Nanoscale* **2022,** 14, 8501.
